# Blocking extracellular activation of myostatin as a strategy for treating muscle wasting

**DOI:** 10.1038/s41598-018-20524-9

**Published:** 2018-02-02

**Authors:** M. Pirruccello-Straub, J. Jackson, S. Wawersik, M. T. Webster, L. Salta, K. Long, W. McConaughy, A. Capili, C. Boston, G. J. Carven, N. K. Mahanthappa, K. J. Turner, A. Donovan

**Affiliations:** 1 Scholar Rock, Inc, Cambridge, MA USA; 2 Present Address: Idexx Laboratories, Westbrook, ME USA; 3 Present Address: Oncorus, Cambridge, MA USA; 4 Present Address: Compass Therapeutics, Cambridge, MA USA

## Abstract

Many growth factors are intimately bound to the extracellular matrix, with regulated processing and release leading to cellular stimulation. Myostatin and GDF11 are closely related members of the TGFβ family whose activation requires two proteolytic cleavages to release the growth factor from the prodomain. Specific modulation of myostatin and GDF11 activity by targeting growth factor-receptor interactions has traditionally been challenging. Here we demonstrate that a novel strategy for blocking myostatin and GDF11, inhibition of growth factor release, specifically and potently inhibits signaling both *in vitro* and *in vivo*. We developed human monoclonal antibodies that selectively bind the myostatin and GDF11 precursor forms, including a subset that inhibit myostatin proteolytic activation and prevent muscle atrophy *in vivo*. The most potent myostatin activation-blocking antibodies promoted robust muscle growth and resulted in significant gains in muscle performance in healthy mice. Altogether, we show that blocking the extracellular activation of growth factors is a potent method for preventing signaling, serving as proof of concept for a novel therapeutic strategy that can be applied to other members of the TGFβ family of growth factors.

## Introduction

Muscle atrophy is associated with a variety of clinical conditions, including diseases of denervation, genetic disorders, cachexia syndromes, and disuse. In these patients, reduced muscle strength and function are highly disabling and lack adequate treatments, often undermining successful management of the primary condition. Loss of muscle strength and mass also occurs as a natural process of aging and in severe cases is categorized as sarcopenia. While varied in their cause, these indications lead to significant disability, lengthy physical rehabilitation and recovery times, and quality of life impairment.

Despite intense interest, pharmacological interventions for preventing or reversing muscle atrophy remain an unmet medical need. Therapeutics for muscle atrophy currently in preclinical or clinical development include inhibitors of myostatin (also known as Growth and Differentiation Factor 8 or GDF8), a secreted growth factor that negatively regulates muscle mass. Myostatin’s expression is mainly restricted to skeletal muscle, with low levels of mRNA reported in adipose^1^ and cardiac^2^ tissues. Naturally occurring and engineered mutations that reduce myostatin signaling lead to a hypermuscular phenotype^1,3–8^. Conversely, myostatin overexpression in rodents induces a cachexia-like syndrome with muscle wasting and fat loss^9^. Because of the well-established impact of myostatin signaling on muscle mass, a number of agents that target the myostatin signaling pathway have entered the clinic for indications involving muscle atrophy.

Myostatin signals by binding a cell surface receptor complex consisting of a type I receptor (ALK4/5) and a type II receptor (ACTRIIa/b)^1^. To date, all of the anti-myostatin drug candidates in early-stage clinical studies block the interaction between mature myostatin and its receptors through antibodies, ligand traps, or overexpression of natural inhibitors such as Follistatin^10–26^. However, because the receptor recognition surfaces of mature myostatin and other Transforming Growth Factor β (TGFβ) family members share a high degree of similarity^27–29^, many of these myostatin-targeted biologics cross-react with other members of the TGFβ family (most commonly GDF11 or Activin A). In all of these cases, while promising results have been reported, it is too early to fully appreciate both their efficacy in patients and potential clinical toxicities associated with their lack of target specificity.

**Figure 1:**
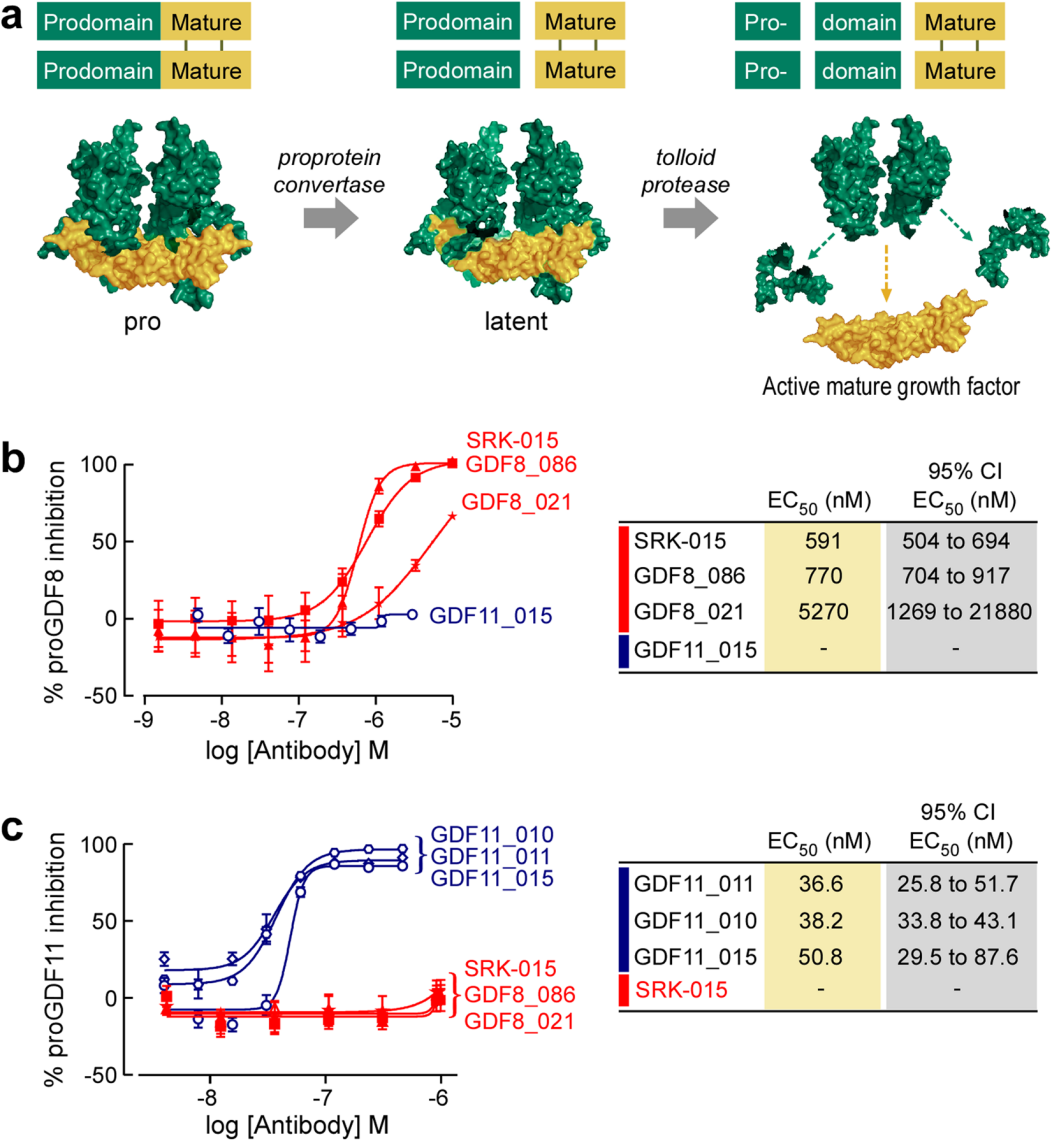
Scholar Rock antibodies specifically block proteolytic activation of myostatin and GDF11. (**a**) Activation of myostatin requires two distinct proteolysis events, generating three major myostatin species. The first cleavage step of proGDF8/proMyostatin (and proGDF11) is carried out by a proprotein convertase to produce the latent complex. Activation and release of the mature growth factor is accomplished after cleavage by an additional protease from the BMP/tolloid family. (**b**,**c**) Precursor-specific antibodies raised towards myostatin (GDF8_021, GDF8_086, and SRK-015) or GDF11 (GDF11_010, GDF11_011, and GDF11_015) were evaluated in myostatin (**b**) and GDF11 (**c**) activation assays. Results were compared to control reactions to calculate the fraction of proMyostatin or proGDF11 activated in the assay. Antibody function was then expressed as the percent inhibition of signaling.

Here we demonstrate the efficacy of a novel alternative therapeutic approach to preventing growth factor engagement with cell surface receptors: blocking growth factor maturation from its autoinhibited precursor forms. Like other TGFβ family members, myostatin and its close homolog GDF11 are synthesized as inactive precursor polypeptides (here termed proMyostatin and proGDF11). Mature growth factor release from the unprocessed precursor is regulated through two discrete protease cleavage events (Fig. 1a). ProMyostatin and proGDF11 are cleaved by proprotein convertases, such as Furin or Proprotein Convertase Subtilisin/Kexin type 5 (PCSK5), that recognize a conserved RXXR site between the prodomain and mature growth factor^9,30–32^. This cleavage produces an inactive latent complex, here termed latent myostatin or latent GDF11, with two prodomains remaining associated with the growth factor dimer, preventing receptor binding.

Release of the active growth factor is accomplished through a separate prodomain cleavage by a protease from the BMP/tolloid family, such as Tolloid-like Protein 2 (TLL-2) or Bone Morphogenetic Protein 1 (BMP1)^30,33^ (Fig. 1a). This additional cleavage releases the mature growth factor from the latent complex, allowing it to bind to its receptor and activate signaling via the SMAD2/3 pathway. In muscle, this ActRIIb-induced signaling inhibits protein synthesis and enhances protein degradation pathways leading to muscle atrophy^34,35^.

*In vivo*, myostatin is detected both in circulation, where it exists predominantly in its latent form^9,36–38^, and in muscle where it resides primarily as unprocessed proMyostatin^39^. Notably, myostatin precursor forms are significantly more abundant in these compartments than the active mature growth factor^9,36,37,39,40^. Here we show that pro- and latent myostatin reside predominantly in the extracellular space within the muscle fiber, in close proximity to the cell surface. In addition, we show that myostatin precursors alter their distribution during glucocorticoid-induced atrophy, suggesting that myostatin processing plays a biologically important role in muscle atrophy.

We also demonstrate that highly specific TGFβ family growth factor regulation can be achieved by modulating extracellular activation, a fundamentally distinct approach from blocking receptor/growth factor interactions. We developed fully human monoclonal anti-proMyostatin and anti-proGDF11 antibodies that potently inhibit their target by blocking growth factor release from the prodomain and, consequently, signaling. Because the prodomains share a much lower degree of conservation (47% identity between myostatin and GDF11 prodomains vs. 90% in the mature domains), this approach allows greater control of antibody specificity. These precursor-directed antibodies were specific for their intended targets *in vitro*. *In vivo*, myostatin-specific activation-blocking antibodies were able to prevent atrophy in a corticosteroid atrophy model, and also increased muscle mass and function in healthy animals. Taken together, these data suggest that inhibition of extracellular activation is a potent and highly selective method for inhibiting TGFβ family signaling.

## Results

### Specific modulation of the activation of myostatin and GDF11

Most growth factor-targeted therapeutic antibodies block the growth factor-receptor interaction. However, release of mature growth factor from the prodomain is tightly regulated in many TGFβ family members, offering an alternative strategy to modulate signaling. We therefore developed a novel phage display approach, using recombinant proMyostatin, latent myostatin and proGDF11 as antigens to screen for antibodies against the pro- and latent forms of myostatin and GDF11. Antibodies were then counterscreened against the mature growth factors to identify fully human monoclonal antibodies that specifically recognize the pro- and latent forms of myostatin or GDF11 without recognizing the mature growth factors. Table 1 summarizes data for six of the monoclonal antibodies identified in our screen: three with specificity for myostatin precursors and three with specificity for GDF11 precursors.

**Table 1:** Binding and activity profile of anti-proGDF11 and anti-proGDF8 antibodies.

	Kd Human proMyostatin* (nM)	Kd Human Latent Myostatin (nM)	Kd Human Mature Myostatin (nM)	Kd Human pro + Latent GDF11 (nM)	Kd Human Latent GDF11 (nM)	Kd Human Mature GDF11 (nM)	EC50 proMyostatin Activation Assay (nM)	EC50 proGDF11 Activation Assay (nM)
SRK-015	2.9	2.4	^2^n.b.	^2^n.b.	^1^n.b.	^1^n.b.	591	none
GDF8_086	0.3	0.5	^2^n.b.	^1^n.b.	^1^n.b.	^1^n.b.	770	none
GDF8_021	3.52	^3^n.b.	^2^n.b.	^1^n.b.	^1^n.b.	^1^n.b.	5270	none
GDF11_010	^1^n.b.	^1^n.b.	^1^n.b.	0.91	0.27	^1^n.b.	none	32
GDF11_011	^1^n.b.	^1^n.b.	^1^n.b.	1.08	0.31	^1^n.b.	none	36.6
GDF11_015	^1^n.b.	^1^n.b.	^1^n.b.	<1^#^	<1^#^	^1^n.b.	none	50.8

Summary of data compiled by bio-layer interferometry (ForteBio Octet), ELISA assays as indicated (method described in the Supplementary methods), and in a luciferase reporter-based activation assay for either human proGDF11 or human proGDF8 (described in methods). “None” indicates the activity data was unable to be fit. The highest concentration of antibody used is indicated when no binding event was observed.

^*^Human proMyostatin in these experiments contains approximately 10–15% latent myostatin.

^#^Tight binder, Kd not resolved by octet.

^1^n.b.: not detected by ELISA.

^2^n.b.: no binding detected at 333 nM Ab.

^3^n.b.: no binding detected at 200 nM Ab.

To evaluate whether the antibodies inhibit proMyostatin or proGDF11 activation, we developed an assay in which recombinant proMyostatin or proGDF11 were incubated with a tolloid protease (mTLL2 or BMP-1) and a proprotein convertase (Furin or PCSK5) (Fig. 1a.). Following proteolysis the activity of released growth factor was measured on 293 T cells expressing a SMAD2/3-dependent luciferase reporter. Because uncleaved recombinant proMyostatin and proGDF11 cannot induce signaling, antibodies that inhibit protease activation and growth factor release will result in reduced luciferase expression from the reporter cell line.

We identified multiple antibodies that specifically blocked proteolytic activation of either proMyostatin or proGDF11 (Fig. 1b,c, Table 1). None of the antibodies cross-reacted between myostatin and GDF11 in binding or inhibition of signaling, indicating the high specificity of this targeting strategy. It should be noted that a high concentration of precursor (proMyostatin in particular) is needed to drive the proteolysis reaction to completion in this assay, leading to artificially high EC50 values, even with the high affinity antibodies described here. Overall, these data demonstrate selective inhibition of myostatin and GDF11 signaling by antibodies that target precursor proteins rather than the mature growth factor domains.

We employed an expanded panel of myostatin precursor-specific antibodies (identified in our screen) to ask whether inhibition of myostatin activation is necessary and sufficient to inhibit myostatin signaling *in vitro* and *in vivo*. We chose ten antibodies with a wide range of binding profiles for this experiment as assessed by epitope binning and affinity measurements towards murine GDF8 proteins (Table 2 and Supplementary Fig. S1). These antibodies exhibited binding to 1) all three myostatin forms, (GDF8-013); 2) pro- and latent- myostatin (SRK-015, GDF8-030, GDF8-086, GDF8-040, GDF8-008, GDF8-062); 3) only proMyostatin (GDF8-021, GDF8-020); and 4) latent and mature myostatin (GDF8-083). Importantly, none of these antibodies recognized GDF11 and all displayed high affinity to their targets. This panel included SRK-015, an optimized version of the best myostatin selective antibody identified in our screen (SRK-015P, P stands for “parental”). SRK-015 was engineered by replacing five resides in the variable domain of SRK-015P outside of the complementarity-determining region (the residues which contact the myostatin precursors). SRK-015 showed a slight increase in affinity for myostatin precursors (compare Tables S1 and S2).

**Table 2:** Binding and activity profile of ten myostatin precursor-binding antibodies and two control antibodies to murine GDF8 and GDF11.

	Kd Murine proMyostatin (nM)	Kd Murine Latent Myostatin (nM)	Kd Murine Mature Myostatin (nM)	Kd Murine pro + Latent GDF11 (nM)	Kd murine Latent GDF11 (nM)	Kd Murine Mature GDF11 (nM)	EC50 Activation Assay (nM)	Epitope bin
SRK-015	2.3 ± 0.069	2.0 ± 0.058	^1^n.b.	n.b.	n.b.	n.b.	174	1 A
GDF8_086	3.7 ± 0.121	4.2 ± 0.130	^1^n.b.	n.b.	n.b.	n.b.	373	1 A
GDF8_040	1.6 ± 0.101	1.8 ± 0.106	^1^n.b.	n.b.	n.b.	n.b.	1380	1 A
GDF8_030	32.9 ± 1.647	28.4 ± 1.360	^1^n.b.	n.b.	n.b.	n.b.	555	1B
GDF8_008	8.5 ± 1.491	7.8 ± 1.190	^1^n.b.	n.b.	n.b.	n.b.	none	1 C
GDF8_062	3.9 ± 0.137	1.9 ± 0.074	^1^n.b.	n.b.	n.b.	n.b.	none	2
GDF8_021	1.4 ± 0.222	n.b.	^1^n.b.	n.b.	n.b.	^2^n.b.	1603	3
GDF8_020	4.3 ± 0.126	n.b.	^1^n.b.	n.b.	n.b.	n.b.	none	4
GDF8_083	n.b.	18.8 ± 1.343	2.1 ± 0.129	n.b.	n.b.	^2^n.b.	none	5 A
GDF8_013	4.7 ± 0.706	11.0 ± 0.353	9.5 ± 0.074	n.b.	n.b.	n.b.	none	5B
GDF8-C1	n.b.	1.0 ± 3.392	0.047 ± 0.040	n.b.	n.b.	n.b.	NA	5 C
GDF8-C5	n.b.	<1^#^	0.9 ± 0.171	5.6 ± 0.179	3.0 ± 0.112	<1^#^	NA	6

Summary of data compiled by bio-layer interferometry (ForteBio Octet), and in the luciferase reporter-based activation assay (described in methods). Also summarized in this table are the results from cross-blocking experiments (see Supplementary data).

^#^Tight binder, Kd not resolved by octet.

n.b.: no binding detected at 200 nM Ab concentration.

^1^n.b.: no binding detected at 333 nM Ab concentration.

^2^n.b.: non-specific binding detected at 200 nM Ab concentration.

NA: not applicable.

None: EC50 curve was unable to be fit (e.g. no inhibition).

**Figure 2:**
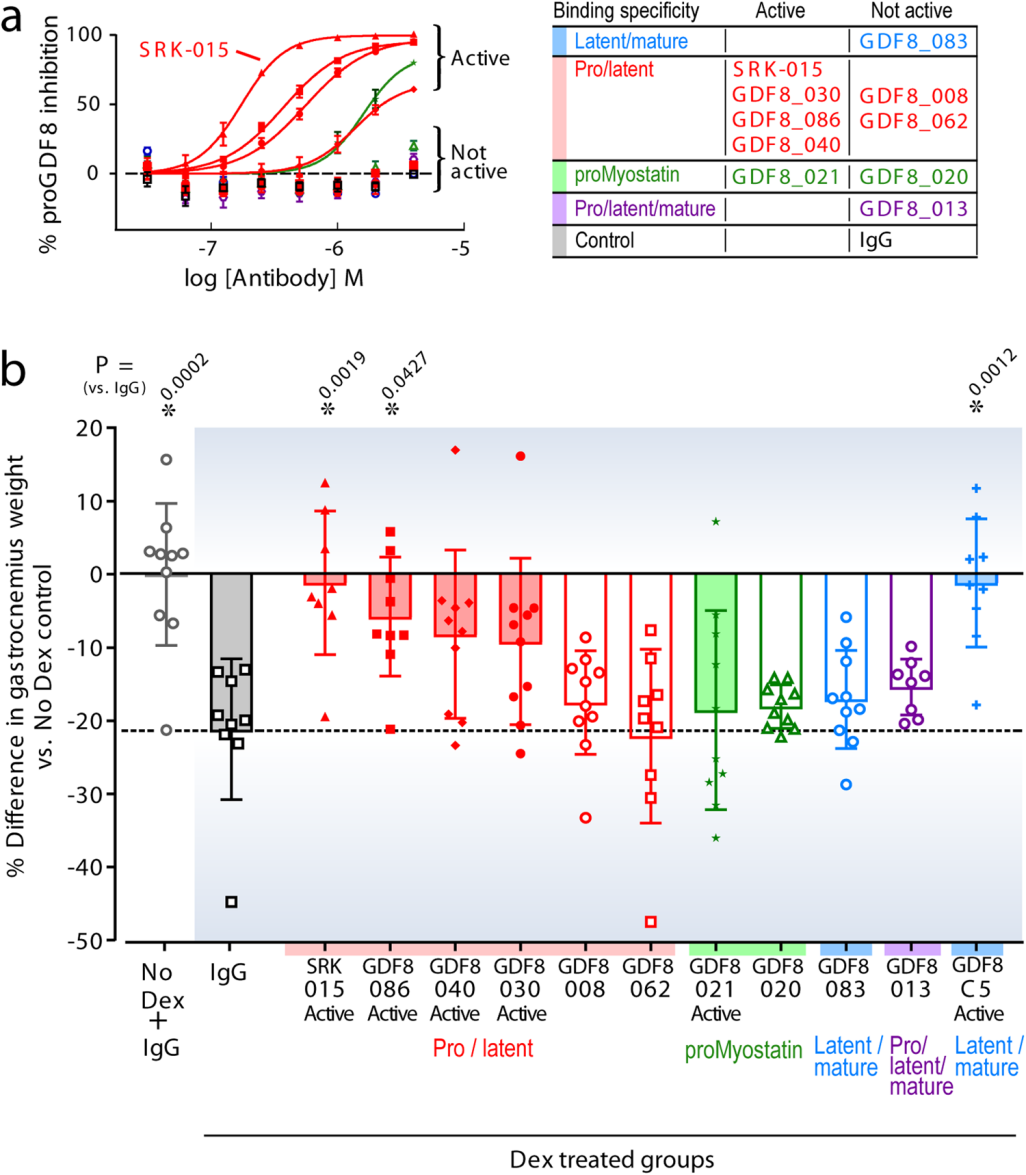
Binding to myostatin precursors and blocking activation prevents muscle atrophy *in vivo*. (**a**) A panel of ten diverse myostatin-precursor specific antibodies were evaluated in the myostatin activation assay. 200 nM murine proMyostatin (proGDF8) was preincubated with increasing concentrations of the test antibodies and signaling inhibition is expressed as a percent inhibition. EC50 values are reported in Table 2. (**b**) Evaluation of the efficacy of myostatin precursor-recognizing antibodies in a skeletal muscle atrophy model. Animals were administered dexamethasone in their drinking water, and dosed with 20 mg/kg of test antibodies once per week for two weeks. The percent difference of the gastrocnemius muscle (versus the mean of the IgG control treated animals without dexamethasone, No Dex) is plotted. Individual data points (n = 8–10 animals) along with means +/− standard deviations are shown. Group means were compared by one-way ANOVA followed by a Holm-Sidak test. Calculated P values for test article compared to the IgG control dosed in combination with dexamethasone are shown.

Three antibodies in the panel (SRK-015, GDF8_086, and GDF8_030 dose-dependently inhibited myostatin release in the myostatin activation assay, with calculated EC50 values ranging from 174–555 nM, close to the concentration of proMyostatin in the assay (200 nM) (Fig. 2a and Table 2). Two others, GDF8_021 and GDF8_040, weakly inhibited proMyostatin activation with EC50 values greater than 1 μM. The remainder of the precursor-binding antibodies in the panel failed to demonstrate inhibitory activity in this assay. These data demonstrate that high affinity binding alone to myostatin precursors is insufficient to block activation *in vitro*.

To compare direct receptor blockade to inhibition of activation, we assessed the ability of SRK-015, GDF8_021, and GDF8-C5^41,42^ to inhibit signaling of mature myostatin. GDF8-C5^41,42^ binds to the latent and mature species of both myostatin and GDF11 and directly inhibits mature growth factor signaling on reporter cells (see Supplementary Fig. S2 and Table 2). This control antibody (along with two others used throughout this manuscript) was produced based on publically available sequences of monoclonal antibodies which target the mature myostatin growth factor and are, or have been, in clinical development. While GDF8-C5 potently inhibited the ability of mature myostatin to signal in the cell based assay, neither SRK-015 nor GDF8_021 inhibited signaling of the mature growth factor (see Supplementary Fig. S2 and Table 2).

### Blockade of myostatin activation prevents dexamethasone-induced muscle atrophy

The panel of antibodies described above allowed us to evaluate the significance of blocking extracellular activation *in vivo*, as each blocking antibody in the panel has a partner antibody with a similar binding profile that lacks the capacity to inhibit myostatin activation (all supporting data summarized in Table 2). Such experimental design allows us to establish whether inhibition of activation is necessary for blocking myostatin signaling, or if binding to precursor forms is sufficient to block myostatin signaling *in vivo*.

For this experiment we chose a dexamethasone-induced muscle atrophy model due to its relevance to myostatin signaling and human disease. Elevated circulating glucocorticoids are associated with a number of muscle wasting diseases^43–46^. In addition, therapeutic use of corticosteroids can lead to induction of muscle atrophy, which was recently explored in rodent models^47^. A number of glucocorticoid receptor target genes have been described to mediate catabolic responses in muscle such as DNA damage-inducible transcript 4 protein (DDIT4/REDD1), Krueppl-like factor 15 (KLF15), and the E3 ubiquitin ligases Murf1/TRIM63 and F-box only protein 32 (FBXO32/Mafbx/atrogin-1)^48,49^. Importantly, myostatin is thought to be a key mediator of catabolic effects of steroid hormones through glucocorticoid responsive elements in its promoter^50–53^.

Muscle atrophy in male C57BL/6 mice was induced by two weeks of dexamethasone treatment. Starting on day 1 of the study, mice were given either normal drinking water (vehicle) or water containing dexamethasone. Concurrent with dexamethasone treatment, mice were administered anti-Myostatin antibodies (Fig. 2) and a negative control antibody (IgG) once weekly (20 mg/kg) by intraperitoneal (IP) injection. Muscle atrophy was monitored during the study by measurement of changes in lean mass from baseline, measured by QNMR at baseline, 1 week and 2 weeks post start of dexamethasone treatment (see Supplementary Fig. S3a) and by measurement of muscle weights at the end of the 2 week study (Fig. 2 and Supplementary Fig. S3b).

Dexamethasone treated animals that received IgG control antibody showed significant atrophy in gastrocnemius muscles (~20%) compared to controls that did not receive dexamethasone (No Dex) (Fig. 2b, black squares, all calculated P values are reported in Table S4). In contrast, both the GDF8-C5 positive control antibody and two of the five myostatin activation-blocking antibodies (SRK-015 and GDF8_086) showed statistically significant protection against gastrocnemius muscle atrophy (vs. IgG control, P = 0.0012, 0.0019 and 0.0427 respectively) with no difference in muscle weights as compared to the healthy control (vs. No Dex) (Fig. 2b). Two additional activation-blocking antibodies (GDF8_030 and GDF8_040) showed trends towards protection but no significant difference from the IgG control. One antibody in a unique epitope class with weak blocking activity *in vitro*, GDF8_021, showed no significant effect on atrophy. Similar results were observed with rectus femoris muscle weights and total body lean mass (see Supplementary Fig. S3). Overall, antibodies that bind pro/latent myostatin (but do not inhibit its activation) all failed to protect against atrophy.

These data confirm that pro- and latent myostatin-specific antibodies that prevent growth factor release can inhibit myostatin signaling *in vivo*. Furthermore, the equivalent potency of SRK-015 and GDF8-C5 suggest that blocking myostatin proteolytic cleavage is as effective as conventional blockade of myostatin receptor/ligand binding.

### SRK-015 uniquely targets the proteolysis of the pro- and latent forms of myostatin

As shown in Table 3, our most potent myostatin precursor-specific antibody, SRK-015, and its parental version, SRK-015P bound robustly to proMyostatin and latent myostatin, and no binding could be detected against the TGFβ family members GDF11, Activin A, BMP9 and 10, or TGFβ1. This binding profile is unique when compared to two antibodies that bind and directly inhibit the growth factor domain (see Supplementary Fig. S2 and Table 3 produced based on publically available sequences from clinical-stage antibodies). One, GDF8-C1^54^, recognized the latent and mature forms of myostatin. The second control antibody, GDF8-C3^55^, displayed a broader recognition profile, binding to not only GDF11 and myostatin, but also to Activin A and BMP10, demonstrating the lack of specificity which arises from targeting the mature growth factor.

**Table 3:** SRK-015/SRK-015P uniquely targets Myostatin precursors.

	*SRK-015*	*SRK-015P*	*GDF8-C1*	*GDF8-C3*
ProMyostatin	2.9 E-09	6.2 E-09	n.b.	n.b.
Latent myostatin	2.4 E-09	9.1 E-09	<1.0E-12^#^	n.b.
Myostatin growth factor	n.b.	n.b.	<1.0E-12^#^	<1.0E-12^#^
ProGDF11	n.b.	n.b.	n.b.	n.b.
GDF11 growth factor	n.b.	n.b.	n.b.	<1.0E-12^#^
ProActivin A	n.b.	n.b.	n.b.	6.22E-09
Activin A growth factor	n.b.	n.b.	n.b.	4.94E-09
BMP 9 growth factor	n.b.	n.b.	n.b.	n.b.
BMP10 growth factor	n.b.	n.b.	n.b.	1.90E-08
TGFB1 growth factor	n.b.	n.b.	n.b.	n.b.

Kd values (M) are reported for indicated antibody/antigen pairs (measured with biolayer interferometry). All proteins listed in this table were human versions.

^#^Tight binder, Kd not resolved by octet.

n.b.: no binding detected at 333 nM antibody concentration.

**Figure 3:**
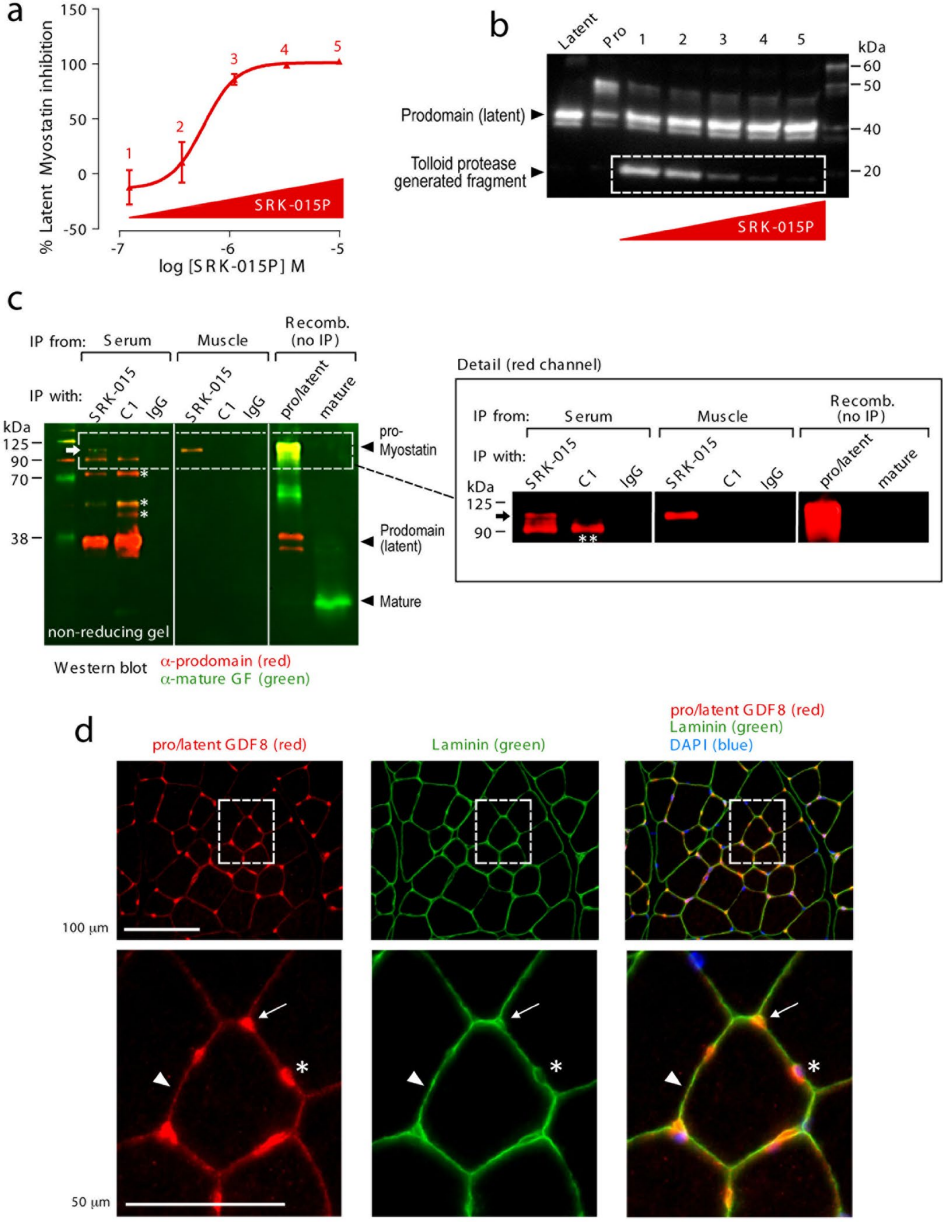
SRK-015/SRK-015P blocks tolloid protease cleavage of the prodomain and binds endogenous proMyostatin in the muscle. (**a**) 500 nM human latent myostatin preincubated with increasing amounts of SRK-015P, were analyzed in a myostatin activation assay. Concentrations of antibodies in each sample were 0.12 μM (1), 0.37 μM (2), 1.1 μM (3), 3.3 μM (4), and 10 μM (5). The EC50 was 581 nM (95% CL 344–982 nM). (**b**) Samples in (**a**) were run on reducing SDS-PAGE and probed by western blot with an antibody recognizing the prodomain of myostatin. An ~18 kDa band (white box), consisting of the C-terminal cleavage fragment of the prodomain generated after tolloid cleavage, decreased proportionally with increasing doses of SRK-015P. (**c**) Non-reducing western blot for prodomain (red) and the mature growth factor (green) following immunoprecipitation with indicated antibodies. Separate red and green images for the entire gel are shown in Supplementary Fig. S5a. Lanes loaded with recombinant protein controls show the migration of mature myostatin, proMyostatin and the myostatin prodomain (latent myostatin) on the gel, highlighted by black arrowheads. In serum, both SRK-015 and GDF8-C1 bind to latent myostatin (prodomain band) and multiple partially processed precursors (asterisks), however only SRK-015 recognized proMyostatin (top band). In muscle, only SRK-015 precipitated a myostatin protein- proMyostatin (black arrow). (**d**) Cross sections of tibilias anterior muscle probed with anti-pro/latent myostatin (red) and anti-laminin (green) and counterstained with DAPI (blue). Pro/latent myostatin and laminin colocalize in the interstitial space at muscle fiber vertices (arrows), between muscle fibers (arrow heads), and around interstitial nuclei (asterisk).

We next explored the mechanism of SRK-015 and SRK-015P action in more detail. Importantly, SRK-015P inhibited activation of both latent myostatin and proMyostatin, suggesting that the furin cleavage step is not significantly affected by antibody binding (Figs 1b, 2a and 3a). To test whether SRK-015/SRK-015P blocks tolloid protease cleavage, latent myostatin proteolysis was analyzed by tracking a myostatin prodomain fragment (white box, Fig. 3b) that is generated by TLL-2 cleavage. SRK-015P (and SRK-015, data not shown) dose-dependently inhibited generation of this fragment, corresponding with signaling inhibition in the activation assay (Fig. 3a,b). These data suggest that SRK-015P and SRK-015 block myostatin activation by inhibiting prodomain cleavage by tolloid family proteases.

### proMyostatin, the major form of myostatin in the muscle, is stored in the extracellular space

As SRK-015 binds both the pro and latent forms of myostatin, we investigated the abundance of these proforms in mouse serum and skeletal muscle. Our goal was to establish whether these forms of myostatin are present in compartments where they can be bound and inhibited by the antibody. First we confirmed by western blot that the predominant form of myostatin in serum is latent, while proMyostatin is the primary form in muscle (see Supplementary Fig. S4), consistent with previous reports^9,36,37,39^. We next performed immunoprecipitation experiments using non-reducing SDS-PAGE to separate precursor myostatin forms. Figure 3c demonstrates that SRK-015 bound proMyostatin, latent myostatin and other partially processed forms of myostatin in serum and proMyostatin in muscle (for raw data see Supplementary Fig. S5a).

The control antibody GDF8-C1, chosen specifically for this experiment because it bound to latent and mature myostatin *in vitro* (Table 2), also immunoprecipitated partially processed myostatin precursors in serum, but no interactions with proMyostatin homodimers were detected in either muscle or serum. We note that an antibody with the same sequence as GDF8-C1 was previously reported to immunoprecipitate proMyostatin from serum^10^, and, consistent with this, we detected a prominent ~90 kDa band that was immunoprecipitated from serum by both antibodies (double asterisk, Fig. 3c). This band, however, was smaller than proMyostatin found in skeletal muscle (marked by the arrow, detailed blow-up in Fig. 3c), and may therefore be a partially processed precursor found in serum but, notably, not in muscle.

The above data suggest that the majority of myostatin found in the muscle is stored as proMyostatin, but this data cannot discriminate between intracellular and secreted stores of proMyostatin. To address this, we performed immunofluorescence on tibialis anterior muscle from healthy mice using GDF8_086, which specifically detects pro- and latent myostatin (Tables 1 and 2, see Supplementary Fig. S6a). Co-staining with laminin, an extracellular matrix marker, demonstrated that the majority of myostatin precursors detected in muscle are interstitial and around interstitial nuclei, with little signal detected intracellularly (Fig. 3d). Co-staining with the vascular marker CD-31 (see Supplementary Fig. S6b) suggested that pools of extracellular myostatin precursors are in close proximity to the circulatory system. Taken together, our data strongly suggest that proMyostatin lies dormant in the extracellular space in healthy muscle and that SRK-015 uniquely recognizes the major forms of myostatin found in both muscle (pro) and serum (latent).

### Myostatin precursor redistribution during atrophy

**Figure 4:**
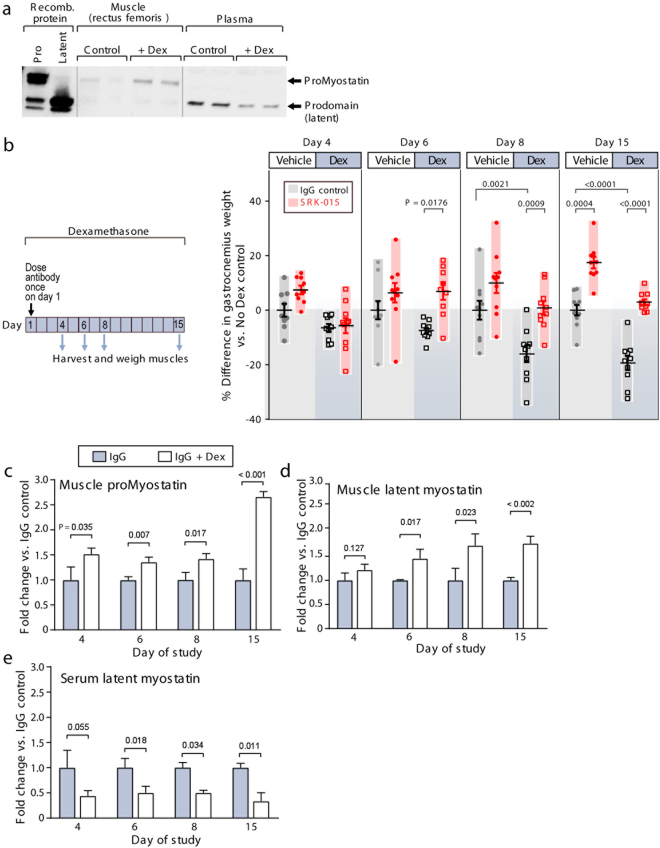
Myostatin precursors redistribute during atrophy. (**a**) Western blot using a polyclonal antibody raised to the prodomain of myostatin. Recombinant protein controls (proMyostatin and latent myostatin) are used to visualize the migration of proMyostatin and myostatin prodomain bands. In muscle from a pilot experiment in which mice were administered dexamethasone for 15 days, proMyostatin levels increase in muscle, while the levels of latent myostatin in plasma (inferred from the prodomain band) decrease. (**b**) In a follow-on experiment, animals were administered either vehicle (No Dex) or dexamethasone in their drinking water for two weeks, and given a single 20 mg/kg dose of test antibodies (SRK-015 or IgG control) at day 1. Differences in gastrocnemius muscle weights (expressed as percent difference from the mean of the IgG (no Dex) control group) are shown for days 4, 6, 8 and 15. Individual data points (n = 8–10 animals) along with means +/− standard deviations are shown. Group means were compared by one-way ANOVA followed by a Holm-Sidak test. (**c**–**e**) Quantitation of proMyostatin and latent myostatin levels in murine muscle and serum at 4, 6, 8, and 15 days following either dexamethasone or vehicle administration. For all data presented, a minimum of three biological replicates were measured to generate the presented average values, and error bars on all graphs represent standard deviations. Statistical significance was determined by t test (two-tailed, homoscedastic).

Western blot analysis of animals treated for 15 days with dexamethasone (Fig. 4a) suggested significant alteration of pro- and latent myostatin in the muscles of dexamethasone-administered mice. To further explore the effects of inhibition of myostatin activation in muscle atrophy, we again employed the dexamethasone-induced muscle atrophy model (described above). In this experiment, however, mice were given either normal water (Vehicle) or dexamethasone-spiked water (Dex) and administered a single 20 mg/kg injection of IgG control antibody or SRK-015. Cohorts of animals were then sacrificed on days 4, 6, 8, and 15 and muscle weights determined.

By day 8, gastrocnemius weights of IgG control mice administered dexamethasone were significantly smaller than mice given normal water (P = 0.0021), signifying that these mice had undergone muscle atrophy, an effect that was even more pronounced at day 15 (P < 0.0001, Fig. 4b). In contrast, animals treated with a single injection of SRK-015 were completely protected from dexamethasone-induced muscle loss, with no difference in muscle weight compared to IgG control mice given normal water (Fig. 4b). In addition to preventing atrophy in dexamethasone treated mice SRK-015 induced a significant increase in muscle mass in healthy (non-dexamethasone treated) animals (17.5% increase, Fig. 4b) 15 days after a single dose of antibody. Furthermore, in accordance with the above data, SRK-015 treatment significantly blunted the upregulation of atrogenes caused by dexamethasone treatment on day 4 (see Supplementary Fig. S7). Interestingly, SRK-015 had no effect on myostatin gene expression (see Supplementary Fig. S7).

In order to understand whether the distribution of myostatin precursor forms is altered during atrophy, we examined myostatin precursors in muscle and serum from the above study using a quantitative fluorescent western blot assay. We confirmed the specificity of the assay in both muscle and serum using samples from mice in which the myostatin prodomain was deleted (see Supplementary Fig. S4). Due to a lack of specific reagents, we were not able to quantify the levels of mature myostatin, though ours and others’ data suggest that mature myostatin levels (outside of the latent complexes) are very low^36,39,40^.

Levels of proMyostatin and latent myostatin were quantified relative to untreated controls (n = 3 per group, Fig. 4c–e), with similar results observed in four independent studies (original western blot images from this experiment are shown in see Supplementary Fig. S5b,c). Following 14 days of dexamethasone treatment, we observed an increase in proMyostatin (1.77 × no dex controls) in the muscle and a decrease in latent myostatin (0.34 × no dex controls) in the serum compared to vehicle treated animals (P < 0.001 and <0.002, respectively, Fig. 4c–e). The altered biodistribution of the myostatin precursors suggests an alteration in the myostatin activation pathway. We also observed dexamethasone-induced changes in myostatin precursors occurring prior to or concomitant with statistically significant decreases in muscle weights (between days 4 and 6), suggesting that redistribution of myostatin precursors may be an important driver of reduced muscle mass. Thus, these data reveal an increase in proMyostatin levels at the site of myostatin signaling during muscle atrophy.

We also confirmed target engagement by SRK-015, showing that levels of both pro- and latent myostatin rose significantly in muscle and serum following antibody treatment (see Supplementary Fig. S5b–f). These data indicate significant SRK-015 target engagement by day 4 and that engagement is sustained until at least two weeks post dose. These findings are also consistent with the unique mechanism of SRK-015, which binds the most abundant form of myostatin (proMyostatin) at the site of signaling. This is in contrast to other monoclonal antibodies that bind the mature growth factor domain without recognizing proMyostatin (such as GDF8-C1, Fig. 3c). Thus, to our knowledge, SRK-015 remains the only monoclonal antibody that specifically binds pro- and latent myostatin in the muscle, blocking activation.

### Blockade of myostatin activation enhances muscle function in healthy animals

**Figure 5:**
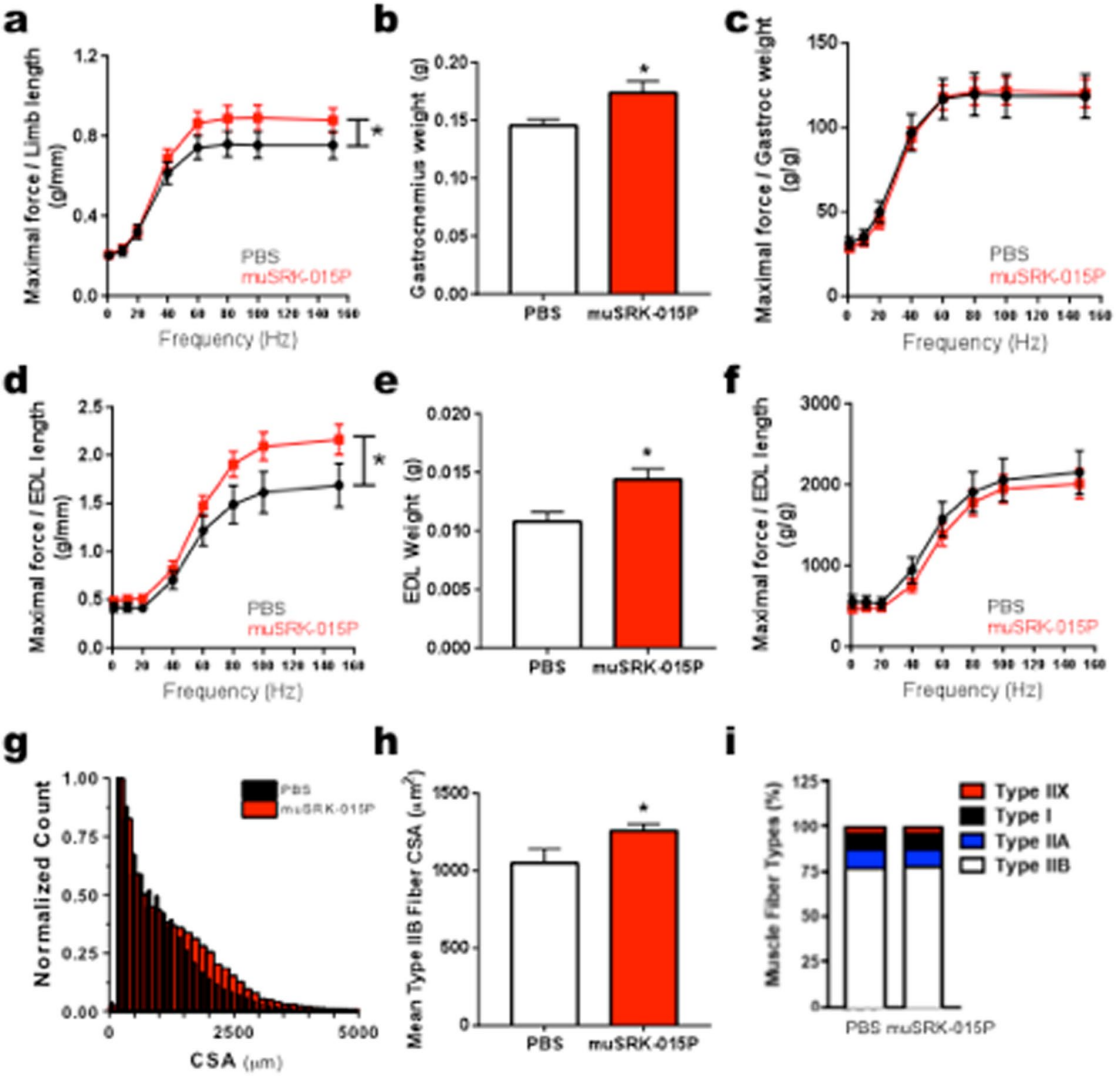
Administration of muSRK-015P in healthy animals enhances muscle function. (**a**–**c**) *In vivo* Plantarflexor functional performance after four weeks of treatment with muSRK-015P. (**a**) *In vivo* maximal force measurements produced from electrical stimulus of the plantarflexor muscle group via the sciatic nerve normalized to limb length. Following 4 weeks of treatment with muSRK-015P, there was an increase of 18% in the maximal force of the plantarflexor group at 100 Hz (Main Effect P = 0.003 by 2-way ANOVA). (**b**) A significant increase in gastrocnemius weight was measured at the end of the study, and when maximal force was normalized to the gastrocnemius weight (**c**) no significant changes were observed. (**d**–**f**) *In vitro* extensor digitorum longus (EDL) muscle function after four weeks of treatment with muSRK-015P. (**d**) Force measurements of the EDL over increasing stimulus frequencies normalized to EDL length. muSRK-015P treatment resulted in a 29% increase in force at 100 Hz (Main effect P = 0.0003 by 2-way ANOVA). (**e**) A significant increase in EDL weight was measured at the end of the study, and when maximal force was normalized to the EDL weight (**f**) no significant changes were observed. (**g**) Increases in cross-sectional area (CSA) were measured following muSRK-015P treatment. (**h**) Significant increases in Type IIB mean fiber area following muSRK-015P treatment (20%, P = 0.011). (**i**) No significant changes in fiber type distribution were observed. Data were analyzed by t-test, Mann Whitney, or Two-way ANOVA as appropriate.

To test whether inhibition of myostatin activation affects muscle performance, we carried out a four-week study in C57BL/6 mice, dosing at 20 mg/kg weekly. We used muSRK-015P, a functionally equivalent, murine constant region chimeric version of SRK-015P (Table S3) to limit the potential immune response that can occur from long term dosing of a human antibody in mice. *In vivo*, animals treated with muSRK-015P generated an 16–18% increase in isometric torque in the plantarflexor group (gastrocnemius, soleus and plantaris muscles, normalized to limb length) at frequencies greater than 60 Hz (main effect P = 0.003) (Fig. 5a). While there was a trend towards increased rate of contraction, this effect was not significant. In addition, no changes were observed in the relaxation rate (see Supplementary Fig. S8a). Because the increase in force was mirrored by a 19% increase in the gastrocnemius weight (P = 0.024, Fig. 5b), there are no differences between groups when force is normalized to muscle weight (Fig. 5c), suggesting that muSRK-015P-induced hypertrophy did not adversely affect muscle quality or excitability.

We next isolated the EDL muscle and measured force *in vitro* to confirm direct effects of myostatin blockade on muscle, independent of nerve function and blood supply. muSRK-015P treatment resulted in a 20–29% increase in maximal force at frequencies greater than 60 hz (main effect P = 0.0003, Fig. 5d) with no alteration in rates of contraction and relaxation (see Supplementary Fig. S8b). Myostatin blockade resulted in a 34% increase in the EDL weight (P = 0.0086, Fig. 5e), such that, again, there were no differences between groups when force was normalized to muscle weight (Fig. 5f).

Histological evaluation of the plantarflexor muscle group revealed a 27% increase in total muscle cross sectional area (P = 0.019, see Supplementary Fig. S8c) and a 14% increase in mean fiber cross sectional area (P = 0.027, see Supplementary Fig. S8d). As expected for a myostatin-specific inhibitor^10^, this increase was underpinned by a 20% increase in Type IIB fiber cross-sectional area (P = 0.011)(Fig. 5g,h), which was independent of any change to relative distribution of fiber types (Fig. 5i) or cross sectional area (CSA) of Type I, IIA, or IIx fibers (see Supplementary Fig. S8e–g). To our knowledge, these data are comparable to or even surpass effects on muscle function reported for other myostatin inhibiting agents^10,56^. Altogether, these data show that inhibition of extracellular myostatin activation promotes a robust anabolic response in the muscle, accompanied by enhanced function with no detrimental side effects.

## Discussion

The data in this manuscript demonstrate a fundamentally novel approach to growth factor inhibition: targeting the prodomain to modulate the *activation* of the mature growth factor rather than by blocking receptor-ligand interactions. This is important, as the high homology of the mature ligands has confounded discovery of truly selective antibodies. Indeed, selective pharmacological modulation of highly related mature myostatin and GDF11 growth factors has proven to be technically challenging. Divergent results have been reported regarding the biological functions of GDF11, with much of the controversy stemming from a lack of reagent specificity^57–60^. We show in this manuscript that by targeting precursor forms of myostatin and GDF11 we were able to generate several highly specific and pharmacologically effective antibodies towards these two homologs. Encouragingly, SRK-015 limited dexamethasone-induced muscle atrophy as effectively as a clinically vetted mature myostatin-targeting antibody (Fig. 2b, compare SRK-015 to GDF8_C5).

Lack of therapeutic specificity extends more broadly within the TGFβ superfamily. Several clinical-stage “anti-myostatins” exhibit cross-reactivity to Activin A, BMPs 9 and 10, and GDF11 (as shown in Tables 2 and 3, for example). Side effects from blocking these growth factors could limit the utility of candidate therapeutics. For example, an early clinical trial of the ActRIIb ligand trap, ACE-031, in Duchenne muscular dystrophy was terminated prematurely due to epistaxis and telangiectasis^26^ attributed to BMP9 or BMP10 reactivity^26,61^. TGFβ family prodomains universally share much less sequence conservation than the mature ligands^29^ and we are currently applying this approach to other members of the family.

Our data confirms and extends previous findings^9,36,37,39^ showing that myostatin is stored extracellularly in its pro- and latent forms *in vivo*. We show that pro- and latent myostatin are the predominant myostatin forms in muscle and serum, respectively. While Anderson *et al*. also demonstrated that some fraction of the proMyostatin pool resides extracellularly, where it can be regulated by extracellular furin cleavage^39^, the relative abundance of intracellular vs. extracellular proMyostatin in muscle tissue remained unclear. By immunofluorescence with a pro/latent-specific antibody, we show here that the bulk of proMyostatin is stored in the sarcolemma-associated extracellular matrix (Fig. 3d). ProMyostatin distribution overlapped with laminin, but was more focal, often detected near junctions between three sarcolemma, suggesting that additional factors may influence proMyostatin extracellular distribution. Indeed the extracellular matrix proteins latent TGFβ binding protein 3 (LTBP3), latent TGFβ binding protein 4 (LTBP4), and perlecan, have been reported to interact with myostatin and/or myostatin precursors^39,62,63^, though the role of these anchoring proteins in modulating signaling is, as yet, unknown.

Our finding that most proMyostatin resides extracellularly in its primary target tissue, muscle, suggests that extracellular activation is a critical step in myostatin signaling. While the follistatins and GASPs (growth and differentiation factor-associated serum proteins 1 and 2) are naturally occurring inhibitors of myostatin and GDF11, these appear to bind a small fraction of mature myostatin in circulation^36,37,39^. Consistent with this hypothesis, we observed changes in the balance of myostatin precursors in a model of muscle atrophy. We found that proMyostatin protein levels were upregulated in muscle by day 4 of glucocorticoid-induced atrophy, while decreased circulating latent myostatin was evident in the same timeframe. Given that changes in proMyostatin levels occurred prior to significant loss of muscle mass, we believe that reduced circulating latent myostatin was not due to decreased muscle mass. We hypothesize instead that changes in serum latent myostatin reflect enhanced myostatin processing in atrophying muscle, underscoring the importance of proteolytic processing in controlling the myostatin signaling pathway.

While circulating myostatin has been examined as a biomarker of muscle atrophy and dysfunction^38,40,58^, our data highlight the importance of understanding myostatin processing and signaling in the muscle as well as in serum, as circulating myostatin levels may not adequately reflect the extent of myostatin signaling in the muscle itself. Quantitation of myostatin by ELISA or immunoaffinity mass spectrometry has been reported^38,40^, but these measurements do not distinguish between free mature myostatin and mature myostatin in latent complexes (bound to the propeptide or other inhibitory proteins). Reagents with specificity for the various forms of myostatin, such as the antibodies described in this study, could be useful in developing truly specific quantitative assays. This, coupled with additional studies to understand myostatin forms in patient samples and preclinical models, will help elucidate the broader role of extracellular processing in myostatin regulation and help to identify patient populations responsive to anti-myostatin therapy.

While our results highlight the therapeutic potential of inhibiting myostatin activation in muscle wasting diseases, an important step towards clinical development is identification of patient populations that would benefit the most from such a therapy. Evaluation in additional disease models, as well as more detailed analysis of the pharmacokinetic and pharmacodynamic properties of SRK-015 is ongoing. In addition, the panel of antibodies that we have developed also affords the opportunity to measure the distribution and dynamics of myostatin precursor forms in healthy and diseased serum and muscle. Such translational insights, complemented by biomarker data previously generated using other assay systems, can further inform the selection of clinical indications in which blockade of extracellular myostatin activation may bring greatest benefit to patients. Based on the highly regulated release of these growth factors in tissue, the novel mechanism of SRK-015 may afford improved safety and/or efficacy profiles compared to antibodies targeting the mature growth factor domain.

Finally, the finding that prodomain-targeted antibodies can modulate signaling of two independent growth factors, myostatin and GDF11, suggests the broader applicability of our approach. We note that these proteins are closely related, and additional investigation is necessary to understand whether our approach can be extended to other TGFβ family members or, more broadly, to prodomains of other signaling molecules. Nevertheless, the data presented here clearly demonstrate the efficacy of our novel approach to inhibit signaling by blocking maturation of a proprotein to its active form, and suggest a potential to selectively modulate a range of growth factor signaling pathways by targeting extracellular activation.

## Materials and Methods

### Antibody discovery

Full-length human and murine Myostatin and GDF11 proteins were stably integrated into FLP-IN^TM^ T-REX^TM^ 293 cells (Life Technologies, Carlsbad, CA) and expressed according to manufacturer’s instructions (Uniprot reference numbers: O14793, O08689, O95390, Q9Z1W4). Fully human anti-myostatin and anti-GDF11 antibodies were identified using a phage library displaying scFvs (single chain variable fragments)^64^. Separate antibody discovery campaigns were run using recombinant human proMyostatin, latent Myostatin, and proGDF11 for both positive and negative selection as appropriate to obtain antibodies with the desired binding profile. Antibodies were cloned and expressed using standard techniques, and binding profiles assessed on a FortéBio Octet QKe in various formats according to manufacturers directions (Supplementary methods). To potentially reduce immunogenicity, SRK-015 was generated from its parental version, SRK-015P, by introduction of five mutations in the variable domain. These antibodies are identical in the complementarity determining regions (CDRs) and have similar affinities and functional activity both *in vitro* and *in vivo*. muSRK-015P is identical to SRK-015P but contains a murine IgG1 constant region instead of a human IgG4 constant region.

### Reporter cell assays

Fully matured growth factors were purchased from R&D systems. Samples of human or murine proMyostatin (200 or 500 nM, as indicated) or human/murine proGDF11 (50 nM) were incubated with supernatants (either singly or in combination as appropriate) collected from stably integrated 293 cells overexpressing a pro-protein convertase (either Furin/PACE3 or PCSK5) and/or a Tolloid protease (either BMP-1 or mTLL2) at 37 °C for 16 hrs. After the proteolysis reaction, Myostatin or GDF11 activity was assessed by adding samples to 293 T cells containing a stably integrated pGL4 plasmid (Promega, Madison, WI) with a promoter comprising SMAD- responsive CAGA sequences. Cells were incubated at 37 °C for 6 hours before detection of luciferase expression using BRIGHT-GLO^TM^ reagent (Promega, Madison, WI) according to manufacturer’s instructions.

Results were used to calculate the percent inhibition for each condition (% inhibition = 100 × ((Reading with test antibody but in the presence of protease – reading without tolloid protease)/(reading without antibody but in the presence of protease-reading without tolloid protease)). EC50 values were calculated in GraphPad Prism 7.0.1 from a four parameter logistic curve. Activation assay data are representative of at least 3 independent experiments, and means and standard deviations of three technical replicates are shown.

### Dexamethasone model

*In vivo* dexamethasone studies were conducted at the Covance Laboratories Inc. Greenfield, Indiana. The Institutional Animal care and Use Committee approved experiments and procedures conformed to their guidelines. Animals were randomly assigned to treatment groups based on body weight at study initiation. Sample sizes (n = 8–10) are standard for detecting statistically different changes in muscle weights of the muscles examined. All data from animals that perished due to dexamethasone toxicity prior to study completion were removed from the analysis. Dexamethasone data are representative of four separate studies for SRK-015 and two studies for SRK-015P. All other antibodies were evaluated once in this model.

Ten male mice (per group, C57BL/6) were enrolled in the studies at 13–14 weeks of age. Starting on day 1 of the study, mice were given either normal drinking water (vehicle) or water containing dexamethasone (Dex, 17.5 mg/L)(Sigma Aldrich, Dexamethasone 21-phosphate disodium salt) treatment for two weeks. In studies with antibody treatments, test articles were administered by intraperitoneal (IP) injection (20 mg/kg) once per week (Fig. 2b) or once (Fig. 4, and see Supplementary Fig. S3) starting at the time of dexamethasone administration. Echo MRI (QNMR) measured body mass composition parameters (fat mass, lean mass and water content) on the indicated days. At necropsy, (on days 4, 6, 8, or 15), animals were sacrificed via CO_2_ overdose and blood was collected via cardiac puncture for serum preparation. Additionally, upon study termination, gastrocnemius and rectus femoris muscles were dissected from both the right and left legs of study mice. All tissues were weighed and then snap frozen for storage at −80 C.

For analysis, weights of the individual muscles from both legs were combined and the average muscle weight in grams was calculated, the average weights were normalized by initial body weight and the percent difference in muscle weight was calculated relative to the mean of the IgG vehicle control group (no Dex). In all experiments, outliers were identified and removed in GraphPad Prism 7.0.1 using the robust regression and outlier removal (ROUT) method (Q = 1%).

### Immunoprecipitation and western blot from homogenized murine muscle and serum

Using standard procedures, clarified supernatants from homogenized muscle tissues or murine serum were analyzed by SDS-PAGE followed by western blot with the following primary antibodies: anti-myostatin prodomain AF1539 (R&D Systems); anti-mature Myostatin ab124621 (Abcam). Immunoprecipitations with SRK-015, IgG control, or GDF8-C1 antibodies from homogenized clarified muscle lysate and murine serum were carried out using the Thermo Scientific Pierce™ Co-Immunoprecipitation Kit according to the manufacturer’s specifications.

For quantitative fluorescent western blots, samples were loaded onto Any kD Mini-PROTEAN TGX Stain-Free Gels (Bio-Rad) and signal was detected by near-IR imaging (Azure c600, Azure Biosystems) and quantified using the western blot analysis tool in AzureSpot software (Azure Biosystems). Band intensity (number of positive pixels per unit area) from pro and latent Myostatin was measured and normalized to total loaded protein per lane. For all data presented, a minimum of three biological replicates were measured to generate the presented average values, and error bars on all graphs represent standard deviations. Statistical significance was determined by t-test (two-tailed, homoscedastic).

### Muscle performance study

The Institutional Animal Care and Use Committee of the Office of Animal Welfare Assurance at the University of Maryland School of Medicine approved muscle performance studies. Experiments conformed to all Association for Assessment and Accreditation of Laboratory Animal Care guidelines. Muscle physiology experiments were conducted once.

Nine-week old male and female C57BL/6 J mice (n = 10) were acclimated for one week and randomized based on body weight, then assigned to treatment groups with approximately equal body weights across all groups. The treatment or controls were administered I.P. via tuberculin syringe weekly. Following 4 weeks of treatment, plantarflexor function was tested *in vivo*, followed by *in vitro* functional testing of the extensor digitorum longus (EDL).

Muscle performance was measured *in vivo* with a 305 C muscle lever system (Aurora Scientific Inc., Aurora, CAN). Anesthesia was accomplished via inhalation (~5% isoflurane, or to effect), and maintained via nose-cone (~2% isoflurane, or to effect). The knee was isolated using a pin partially inserted into the tibial head and the foot firmly fixed to a footplate on the motor shaft. Contractions were elicited by percutaneous electrical stimulation of the sciatic nerve. Optimal isometric twitch torque was determined by increasing the current with a minimum of 30 seconds between each contraction to avoid fatigue. A series of stimulations were then performed at increasing frequency of stimulation (0.2 ms pulse, 500 ms train duration): 1, 10, 20, 40, 60, 80, 100, 150 Hz, followed by a final stimulation at 1 Hz. Following the measurements, the foot was released and the pin removed and the mouse allowed to recover in a separate clean cage prior to returning to its living chamber.

EDL muscle performance was measured *in vitro* with the 305 C muscle lever system adapted with a horizontal perfusion bath. Briefly, the skin on the lower limb was degloved half-length to the knee and the *tibialis anterior* muscle carefully dissected free from the underlying EDL. A silk suture (4-0) was tied to the distal tendon of the EDL and the tendon severed. The muscle was then carefully dissected free from the tibia and adjacent muscles, the proximal tendon visualized and severed. The muscle was placed in an ice-cold physiological buffered solution and a silk suture tied to the proximal tendon. The muscle was placed in the horizontal bath of the 305 C muscle lever system and perfused with physiological buffer oxygenated with 95% O_2_/5% CO_2_ and kept at 37 °C. The sutures were tied to a fixed post on one side, and the lever arm on the other. A series of 1 Hz and 100 Hz field stimulations (0.2 ms pulse, 100 ms duration) at 0.01 Hz frequency were delivered via platinum electrodes flanking the muscle to ensure that the sutures are tight and that the maximal developed force was stable. Once stable, a series of stimulations were performed at increasing frequency of stimulation (1 ms pulse, 250 ms train duration): 1, 10, 20, 40, 60, 80, 100, 150 Hz, followed by a final stimulation at 1 Hz.

Data were analyzed using Aurora Scientific 615 A Dynamic Muscle Analysis Software Suite in high-throughput mode. Each data file was manually inspected to ensure that cursors and fits were assigned properly and corrected when necessary. Data were then grouped and means and standard errors calculated. Force was normalized to limb length defined as the distance from the knee to the bottom of the ankle. Data were compared over time by calculating the % change using standard equations. Statistical analysis was performed using SigmaPlot v11. Data were analyzed using either a Student’s t-test with Mann-Whitney test, a One-way ANOVA or Two-way ANOVA with Holm-Sidak post-hoc when appropriate with an α = 0.05.

### Histology

The plantarflexor muscle group, soleus, gastrocnemius and tibialis anterior muscles frozen for histology were sectioned and stained according to standard protocols. For quantization using fluorescence microscopy, sections were stained with wheat germ agglutinin to determine cross sectional area or antibodies against MyHC-I, MyHC-IIa, or MyHC-IIb (1:20 dilution; Developmental Studies Hybridoma Database) for fiber-type determination. For proMyostatin localization studies, sections were stained with rabbit anti-laminin (Abcam) and GDF8_086 (Scholar Rock). H&E staining was performed according to Treat-NMD protocol MDC1a_M1.2.004.

### Data Availability

Correspondence and requests for reagents described in this manuscript (available via MTA) can be directed at Adriana Donovan, adonovan@scholarrock.com.

## Electronic supplementary material

Supplementary Information

## References

[1] McPherron AC, Lawler AM, Lee SJ (1997). Regulation of skeletal muscle mass in mice by a new TGF-beta superfamily member. Nature.

[2] Rodgers BD (2009). Myostatin represses physiological hypertrophy of the heart and excitation-contraction coupling. J Physiol.

[3] Grobet L (1997). A deletion in the bovine myostatin gene causes the double-muscled phenotype in cattle. Nat Genet.

[4] Kambadur R, Sharma M, Smith TP, Bass JJ (1997). Mutations in myostatin (GDF8) in double-muscled Belgian Blue and Piedmontese cattle. Genome Res.

[5] Mosher DS (2007). A mutation in the myostatin gene increases muscle mass and enhances racing performance in heterozygote dogs. PLoS Genet.

[6] Schuelke M (2004). Myostatin mutation associated with gross muscle hypertrophy in a child. N Engl J Med.

[7] McPherron AC, Lee SJ (1997). Double muscling in cattle due to mutations in the myostatin gene. Proc Natl Acad Sci USA.

[8] Varga L (2003). Mapping modifiers affecting muscularity of the myostatin mutant (Mstn(Cmpt-dl1Abc)) compact mouse. Genetics.

[9] Zimmers TA (2002). Induction of cachexia in mice by systemically administered myostatin. Science.

[10] Latres E (2015). Myostatin blockade with a fully human monoclonal antibody induces muscle hypertrophy and reverses muscle atrophy in young and aged mice. Skelet Muscle.

[11] Padhi D (2014). Pharmacological inhibition of myostatin and changes in lean body mass and lower extremity muscle size in patients receiving androgen deprivation therapy for prostate cancer. J Clin Endocrinol Metab.

[12] Zhang L (2011). Pharmacological inhibition of myostatin suppresses systemic inflammation and muscle atrophy in mice with chronic kidney disease. FASEB J.

[13] Becker C (2015). *Myostatin antibody (LY2495655) in olde*r weak fallers: a proof-of-concept, randomised, phase 2 trial. The Lancet Diabetes & Endocrinology.

[14] Smith RC (2015). Myostatin Neutralization Results in Preservation of Muscle Mass and Strength in Preclinical Models of Tumor-Induced Muscle Wasting. Mol Cancer Ther.

[15] Woodhouse L (2016). A Phase 2 Randomized Study Investigating the Efficacy and Safety of Myostatin Antibody LY2495655 versus Placebo in Patients Undergoing Elective Total Hip Arthroplasty. J Frailty Aging.

[16] Haidet AM (2008). Long-term enhancement of skeletal muscle mass and strength by single gene administration of myostatin inhibitors. Proc Natl Acad Sci USA.

[17] Kota J (2009). Follistatin gene delivery enhances muscle growth and strength in nonhuman primates. Sci Transl Med.

[18] Mendell JR (2015). A phase 1/2a follistatin gene therapy trial for becker muscular dystrophy. Mol Ther.

[19] Zhu Y (2016). LC-MS/MS multiplexed assay for the quantitation of a therapeutic protein BMS-986089 and the target protein Myostatin. Bioanalysis.

[20] Dankbar B (2015). Myostatin is a direct regulator of osteoclast differentiation and its inhibition reduces inflammatory joint destruction in mice. Nat Med.

[21] Wagner KR (2008). A phase I/IItrial of MYO-029 in adult subjects with muscular dystrophy. Ann Neurol.

[22] Holzbaur EL (2006). Myostatin inhibition slows muscle atrophy in rodent models of amyotrophic lateral sclerosis. Neurobiol Dis.

[23] Lach-Trifilieff E (2014). An antibody blocking activin type II receptors induces strong skeletal muscle hypertrophy and protects from atrophy. Mol Cell Biol.

[24] Amato AA (2014). Treatment of sporadic inclusion body myositis with bimagrumab. Neurology.

[25] Cadena SM (2010). Administration of a soluble activin type IIB receptor promotes skeletal muscle growth independent of fiber type. J Appl Physiol (1985).

[26] Campbell, C. *et al*. Myostatin inhibitor ACE-031 treatment of ambulatory boys with Duchenne muscular dystrophy: Results of a randomized, placebo-controlled clinical trial. *Muscle Nerve*, 10.1002/mus.25268 (2016). 10.1002/mus.25268 27462804

[27] Khalil AM (2016). Differential Binding Activity of TGF-beta Family Proteins to Select TGF-beta Receptors. J Pharmacol Exp Ther.

[28] Padyana AK (2016). Crystal structure of human GDF11. Acta Crystallogr F Struct Biol Commun.

[29] Hinck, A. P., Mueller, T. D. & Springer, T. A. Structural Biology and Evolution of the TGF-beta Family. *Cold Spring Harb Perspect Biol* **8**, 10.1101/cshperspect.a022103 (2016). 10.1101/cshperspect.a022103 PMC5131774 27638177

[30] Ge G, Hopkins DR, Ho WB, Greenspan DS (2005). GDF11 forms a bone morphogenetic protein 1-activated latent complex that can modulate nerve growth factor-induced differentiation of PC12 cells. Mol Cell Biol.

[31] Essalmani R (2008). *In vivo* functions of the proprotein convertase PC5/6 during mouse development: Gdf11 is a likely substrate. Proc Natl Acad Sci USA.

[32] Lee SJ, McPherron AC (2001). Regulation of myostatin activity and muscle growth. Proc Natl Acad Sci USA.

[33] Wolfman NM (2003). Activation of latent myostatin by the BMP-1/tolloid family of metalloproteinases. Proc Natl Acad Sci USA.

[34] Sartori R (2009). Smad2 and 3 transcription factors control muscle mass in adulthood. Am J Physiol Cell Physiol.

[35] Trendelenburg AU (2009). Myostatin reduces Akt/TORC1/p70S6K signaling, inhibiting myoblast differentiation and myotube size. Am J Physiol Cell Physiol.

[36] Hill JJ (2002). The myostatin propeptide and the follistatin-related gene are inhibitory binding proteins of myostatin in normal serum. J Biol Chem.

[37] Hill JJ, Qiu Y, Hewick RM, Wolfman NM (2003). Regulation of myostatin *in vivo* by growth and differentiation factor-associated serum protein-1: a novel protein with protease inhibitor and follistatin domains. Mol Endocrinol.

[38] Bergen HR (2015). Myostatin as a mediator of sarcopenia versus homeostatic regulator of muscle mass: insights using a new mass spectrometry-based assay. Skelet Muscle.

[39] Anderson SB, Goldberg AL, Whitman M (2008). Identification of a novel pool of extracellular pro-myostatin in skeletal muscle. J Biol Chem.

[40] Lakshman KM (2009). Measurement of myostatin concentrations in human serum: Circulating concentrations in young and older men and effects of testosterone administration. Molecular and Cellular Endocrinology.

[41] Geertruida M. Veldman, M. V. D., Kening S, Neil M. Wolfman, Kristie Grove Bridges, Anne Field, Caroline Russell, Viia Valge-Archer. Neutralizing Antibodies Against GDF-8 and Uses Therefor. USA patent (2004).

[42] Recommended INN: List 56: Stamulumab. *WHO Drug Information* **20**, 227 (2006).

[43] Barry SC, Gallagher CG (2003). Corticosteroids and skeletal muscle function in cystic fibrosis. J Appl Physiol (1985).

[44] Decramer M, Lacquet LM, Fagard R, Rogiers P (1994). Corticosteroids contribute to muscle weakness in chronic airflow obstruction. Am J Respir Crit Care Med.

[45] Khaleeli AA, Betteridge DJ, Edwards RH, Round JM, Ross EJ (1983). Effect of treatment of Cushing’s syndrome on skeletal muscle structure and function. Clin Endocrinol (Oxf).

[46] Mills GH (1999). Respiratory muscle strength in Cushing’s syndrome. Am J Respir Crit Care Med.

[47] Quattrocelli M (2017). Intermittent glucocorticoid steroid dosing enhances muscle repair without eliciting muscle atrophy. J Clin Invest.

[48] Shimizu N (2011). Crosstalk between glucocorticoid receptor and nutritional sensor mTOR in skeletal muscle. Cell Metab.

[49] Schakman O, Kalista S, Barbe C, Loumaye A, Thissen JP (2013). Glucocorticoid-induced skeletal muscle atrophy. Int J Biochem Cell Biol.

[50] Gilson H (2007). Myostatin gene deletion prevents glucocorticoid-induced muscle atrophy. Endocrinology.

[51] Wang R, Jiao H, Zhao J, Wang X, Lin H (2016). Glucocorticoids Enhance Muscle Proteolysis through a Myostatin-Dependent Pathway at the Early Stage. PLoS One.

[52] Ma K (2001). Characterization of 5′-regulatory region of human myostatin gene: regulation by dexamethasone *in vitro*. Am J Physiol Endocrinol Metab.

[53] Ma K (2003). Glucocorticoid-induced skeletal muscle atrophy is associated with upregulation of myostatin gene expression. Am J Physiol Endocrinol Metab.

[54] Recommended INN: List 75: Trevogrumab. *WHO Drug Information* **30**, 160–161 (2016).

[55] Recommended INN: List 76: Domagrozumab. *WHO Drug Information* **30**, 495–496 (2016).

[56] Apgar JR (2016). Beyond CDR-grafting: Structure-guided humanization of framework and CDR regions of an anti-myostatin antibody. MAbs.

[57] Egerman MA (2015). GDF11 Increases with Age and Inhibits Skeletal Muscle Regeneration. Cell Metab.

[58] Schafer MJ (2016). Quantification of GDF11 and Myostatin in Human Aging and Cardiovascular Disease. Cell Metab.

[59] Harper, S. C. *et al*. Is Growth Differentiation Factor 11 a Realistic Therapeutic for Aging-Dependent Muscle Defects? *Circ Res* **118**, 1143–1150, discussion 1150, 10.1161/CIRCRESAHA.116.307962 (2016). 10.1161/CIRCRESAHA.116.307962 PMC4829942 27034276

[60] Walker, R. G. *et al*. Biochemistry and Biology of GDF11 and Myostatin: Similarities, Differences, and Questions for Future Investigation. *Circ Res* **118**, 1125–1141; discussion 1142, 10.1161/CIRCRESAHA.116.308391 (2016). 10.1161/CIRCRESAHA.116.308391 PMC4818972 27034275

[61] David L, Mallet C, Mazerbourg S, Feige JJ, Bailly S (2007). Identification of BMP9 and BMP10 as functional activators of the orphan activin receptor-like kinase 1 (ALK1) in endothelial cells. Blood.

[62] Lamar KM (2016). Overexpression of Latent TGFbeta Binding Protein 4 in Muscle Ameliorates Muscular Dystrophy through Myostatin and TGFbeta. PLoS Genet.

[63] Sengle G, Ono RN, Sasaki T, Sakai LY (2011). Prodomains of transforming growth factor beta (TGFbeta) superfamily members specify different functions: extracellular matrix interactions and growth factor bioavailability. J Biol Chem.

[64] McCafferty J, Griffiths AD, Winter G, Chiswell DJ (1990). Phage antibodies: filamentous phage displaying antibody variable domains. Nature.

