# Supplementary material for: Blocking extracellular activation of myostatin as a strategy for treating muscle wasting
Source: Sci Rep. 2018 Feb 2;8:2292. doi: 10.1038/s41598-018-20524-9 (PMC5797207; doi:10.1038/s41598-018-20524-9)
Supplement: Supplementary file 1 — Supplementary Information [file 41598_2018_20524_MOESM1_ESM.doc]

Blocking extracellular activation of myostatin as a strategy for treating muscle wasting

M. Pirruccello-Straub^1,2^, J. Jackson, S^1^. Wawersik^1^, M.T. Webster^1^, L. Salta^1,3^, K. Long^1^, W. McConaughy^1,4^, A. Capili^1^, C. Boston^1^, G.J. Carven^1^, N.K. Mahanthappa^1^, K.J. Turner^1^, and A. Donovan^1*^

^1^Scholar Rock, Inc., Cambridge MA

^2^Present Address: Idexx Laboratories, Westbrook, ME

^3^Present Address: Oncorus, Cambridge, MA

^4^Present Address: Compass Therapeutics, Cambridge, MA

^*^Address correspondence to adonovan@scholarrock.com.

Supplementary materials

**Supplementary Methods**

*Recombinant protein expression and purification*

Protein constructs were stably integrated into FLP-IN^TM^ T-REX^TM^ 293 cells (Life Technologies, Carlsbad, CA). Full-length human and murine Myostatin and GDF11 proteins were expressed according to manufacturer’s instructions (Uniprot reference numbers: O14793, O08689, O95390, Q9Z1W4). After five days of expression, culture supernatant was collected and cleared by centrifugation for 10 minutes at 450 x gravity at 4°C. Supernatant was then filtered by passing it through a 0.22 µm pore filter. Filtered supernatant was combined with Tris, NaCl and NiCl_2_ for a final concentration of 50 mM Tris pH 8.0, 350 mM NaCl and 0.5 mM NiCl_2_ and purified by Ni-NTA chromatography (Qiagen) in 20 mM Tris, pH 8.0, 500 mM NaCl and 20 mM imidazole and eluted with 20 mM Tris, pH 8.0, 500 mM NaCl and 300 mM imidazole. The protein was further purified by size exclusion chromatography (SEC) equilibrated with either HBS, or 20 mM Hepes pH 7.5, 500 mM NaCl.  Peak fractions were pooled and concentrated to a concentration of 1-2 mg/mL and aliquots flash-frozen and stored at -80C.

Latent myostatin was produced via *in vitro* cleavage of purified proMyostatin by human Furin protease expressed and purified in house. Latent GDF11 was produced by adding cells overexpressing PCSK5 (a proprotein convertase) to the GDF11 expressing cells and purifying latent GDF11 from the cell supernatants. Purification proceeded as described above. In both cases, the material was >95% latent, with the proteolysis reaction proceeding almost to completion.

To produce murine proMyostatin preparations free from latent material, a chromatography column consisting of the antibody GDF8_C1, which recognizes both latent and mature myostatin, was created. Recombinant murine proMyostatin preparation (containing ~40% latent Myostatin) was applied to the column. Flow-through containing only proMyostatin was then collected and concentrated to 1.9 mg/mL for use in *in vitro* assays. A homogenous preparation of human proGDF8 was purified from stably integrated 293 cells cultured in the presence of 30 mM decanoyl-RVKR-CMV and purified as described above.

Mature GDF8, mature GDF11, mature Activin A, mature BMP9, mature BMP10, and mature TGFβ1 were purchased from R&D systems.

## Antibody purification

Selected scFv sequences were converted to full-length IgG4 antibodies by cloning the variable regions into pTT5 vectors^1^ containing either human IgG4, murine IgG1, human Kappa, murine Lambda, or human Lambda constant regions. Transient expression was then carried out in Expi293 cells (ThermoFisher, Cat #A14527). Briefly, cells were diluted to 3 x 10^5^ cells/mL in Expi293 expression medium (ThermoFisher, Cat# [A1435102](https://www.thermofisher.com/order/catalog/product/A1435102)) and allowed to grow for 3 days (37°C, 8% CO_2_,) to reach an appropriate density (2-2.5 x 10^6^ cells/mL) for transient transfection. Heavy chain and Light chain DNA were combined at a 1:1 ratio (1 µg total DNA/mL of culture) and 0.144% PEI Max 40000 (Polysciences, Cat# 24765) (2 µg/mL of culture) were incubated with Expi293 expression medium, separately, for 5 min at room temperature; then combined and incubated for an additional 8 min. These DNA:PEI Max complexes were added to the Expi293 cells cultures. After 5 days, transiently transfected cultures were then harvested via centrifugation at 4000 rpm for 20 minutes. The supernatants were then sterile filtered and stored at 4°C until ready for purification.

Antibodies were purified using rProtein A Sepharose Fast Flow (GE Healthcare) at 10 mg antibody/mL resin. Briefly, rProtein A columns were first equilibrated with PBS, loaded with clarified and filtered conditioned media, washed with 20 column volumes of PBS, then eluted under acidic conditions using 100 mM Phosphoric Acid, pH 3.0. Fractions containing eluted antibodies were quickly neutralized with 10% volume of 1.6 M HEPES, pH 8.0. NaCl was then added to a final concentration of 100 mM using a 5 M stock solution. Fractions were then analyzed by both SDS-PAGE and analytical size exclusion chromatography (SEC). For analytical SEC, 10-20 μg were injected onto a Superdex 200 5 x 150 using 20 mM Phosphate, 200 mM NaCl, pH 6.8 as the mobile phase. Fractions containing eluted antibodies were then pooled, concentrated to 10-20 mg/ml, then further purified on a preparative Superdex 200 16x60 using 20 mM Citrate, 150 mM NaCl, pH 6.5 as the mobile phase. Eluted fractions were reanalyzed by SDS-PAGE and analytical SEC, pooled and concentrated to 5 mg/mL. Endotoxin was also measured using the Endosafe PTS system (Charles River). All antibodies used in this study were greater than 95% pure with low endotoxin (less than 0.3 EU/mg). Antibodies were then analyzed by SDS-PAGE on 4-20% Tris-Glycine gradient gels (Biorad).

Antibody concentrations were determined by UV absorbance using calculated extinction coefficients based on amino acid sequences.

## Affinity measurements

50 nM test antibody was used to saturate an anti-human Fc capture tip (FortéBio). Initially, all antibodies were tested against a single 200 nM concentration of human and murine proGDF8, latent GDF8, and mature GDF8. If binding was observed, a Kd value was determined by immobilizing the antibody as previously described and using analyte in titration from 200 to 0.82 nM by 3-fold dilutions. The Kd was determined using a global fit with FortéBio data analysis software 8.2. For binding to mature myostatin and GDF11 5 μg/mL of growth factor (R&D systems) was coupled to amine reactive sensor tips (FortéBio) in acetate buffer at pH 5. All antibodies were initially tested at 333 nM for binding to this myostatin-coupled sensor. Antibodies that showed binding were then tested in concentrations ranging from 333 to 1.37 nM by 3 fold dilutions. A global fit was used to determine the Kd of the interaction using FortéBio data analysis 8.2.

Binding profiles for SRK-015, GDF8-C1, and GDF8-C3 were determined using the FortéBio Octet QKe dip and read label free assay system utilizing bio-layer interferometry. In the binding experiments with human proMyostatin (Table 1), 150 nM of test antibody was used to saturate the immobilization sites on human Fc capture tips (FortéBio), and the association and dissociation of 150 nM analyte was evaluated. Human latent myostatin, proGDF11, and proActivin A were biotinylated according to manufacturer’s instructions (Pierce EZ-Link NHS-PEG4-Biotin) and immobilized to streptavidin-coated biosensors (FortéBio Part No: 18-5060). Mature GDF8, mature GDF11, mature Activin A, mature BMP9, mature BMP10, and mature TGFβ1 were directly immobilized to amine reactive second generation biosensors (FortéBio Part No: 18-5092) according to the protocol provided by the manufacturer. All baseline steps, loading of biotinylated antigens, and antibody association and dissociation steps were performed in 1x kinetics buffer containing PBS pH 7.4 with 0.002% tween 20 and 0.01% BSA. Direct coupling of antigens to biosensors was performed in 20 mM acetate buffer pH 5. A shake speed of 1000 was used for each step and a black polypropylene flat bottom 96 well was used. After antigen immobilization and baselining, the biosensors were moved into wells containing 50 μg/mL of SRK-015. Association proceeded for 300 seconds for biotinylated antigens or 600 seconds for the antigens that were directly conjugated to the amine reactive biosensors. Dissociations were then performed in 1x kinetics buffer for at least 600 seconds. Data analysis was performed on ForteBio Data Analysis software version 8.2. The data were processed by aligning the Y-axis to the final 5 seconds of the baseline step, and inter-step correction to the dissociation was performed. Savitsky-Golay filtering was employed. A 1:1 full fitting model considering both association and dissociation was then utilized for the single concentration Kd determination for each antigen antibody/construct pair.

*Gene expression analysis*

Expression of Murf1, Mafbx, Myostatin, and Metallothionein 2 mRNAs was quantitated using the Quantigene Plex Assay (Affymetrix). Probes for Rpl19 and Ppib were included as internal housekeeping controls. Frozen gastrocnemius samples from healthy or dexamethasone treated mice administered a single 20 mg/kg dose of IgG control or SRK-015 were pulverized using a Covaris CryoPrep. Approximately 5 mg tissue powder was homogenized in Quantigene homogenization solution (60 μL/mg tissue) following the manufacturer’s recommended protocol. Briefly, pulverized tissues in homogenization solution were incubated at 65°C for 30 minutes with 1 minute of vortexing every 10 minutes. Samples were centrifuged to remove any debris, and the clarified homogenate was used in the Quantigene assay. The Quantigene plex assay was performed according to the kit protocol. Samples were analyzed on a Luminex MAGPIX instrument with xPONENT 4.2 software. Data were analyzed in Excel. Each sample was normalized to the geometric mean of the Rpl19 and Ppib signals of that sample. To ensure that expression of these housekeeper genes did not vary across treatment groups, Rpl19 and Ppib were also normalized to the geometric mean. The variance of normalized Rpl19 and Ppib across all sample groups was 10-11%.

## Immunoprecipitation from murine serum and muscle

Homogenized muscle lysate was prepared as follows: frozen mouse quadriceps were pulverized using a CryoPrep pulverizer (Covaris, Woburn MA). The pulverized muscle was then resuspended to a concentration of 50 mg/mL in M-Per buffer (ThermoFisher Scientific) with 1x Halt™ Protease and Phosphatase Inhibitor Cocktail without EDTA (ThermoFisher Scientific). The tissue was then crushed using a plastic pestle, (Bio-Plas Cat #4030-PB) and homogenized further with repeated pipetting with a cut-off pipette tip. Muscle samples were then incubated 30 minutes at 4°C with end-over-end rotation. Finally, samples were centrifuged at 16,100 g for 10 minutes to pellet the insoluble fraction. The soluble fraction was aspirated off and used in downstream experiments.

For immunoprecipitation, SRK-015, IgG control, or GDF8-C1 antibodies were covalently conjugated to agarose beads using the Thermo Scientific Pierce™ Co-Immunoprecipitation Kit according to the manufacturer’s specifications. 75 μg of each antibody was conjugated to 50 μL of bead slurry, and 30 μg of antibody was utilized in each immunoprecipitation. The immunoprecipitation was performed against 3 mL of pooled normal mouse serum (Bioreclamation) or 1.05 mL of homogenized soluble mouse quadriceps prepared as described above. Antibody conjugated beads and samples were incubated at 4°C with end-over-end rocking overnight. After incubation, the beads were recovered by passing the entire sample volume through the spin filters included in the co-immunoprecipitation kit using the QIAvac 24 Plus vacuum manifold. (Qiagen) The beads were then washed 3x with 200 μL of IP lysis/wash buffer, and once with 100 μL of 1x conditioning buffer according to the specifications of the kit. Elutions were performed with 50 μL of elution buffer for five minutes and were then mixed with 5µL of 1M Tris, pH 9.5 in the collection tube.

Myostatin species pulled down by the test antibodies were visualized by western blotting utilizing AF1539, (R&D systems) ab124721, (Abcam) Alexa Fluor® 680 AffiniPure Donkey Anti-Sheep IgG (H+L), (Jackson ImmunoResearch) and IRDye® 800CW Donkey anti-Rabbit IgG (H + L) (LI-COR Biosciences). SEA BLOCK blocking buffer was utilized for the blocking and primary antibody incubations.

*Localization of proMyostatin and latent myostatin in muscle sections*

Tibialis anterior (TA) muscles were fixed in ice cold 4% paraformaldehyde (EMS), PBS for 30 min, incubated overnight in 10% sucrose, PBS at 4°C, then incubated overnight in 20% sucrose, PBS. Muscles were then mounted on cork with tragacanth (Sigma) and frozen in liquid nitrogen cooled isopentane (Sigma) for cryosectioning. 10 μm sections of TA muscle were permeabilized with 0.1% Triton-X 100 (Sigma), PBS for 20 minutes, washed once with 0.05% Triton-X 100, PBS (PBS/T), and then incubated in Mouse IgG blocking reagent (Vector Lab) diluted at 1 drop per 1.5 mL PBS/T for 1 h. Sections were washed once with PBS/T and then incubated in normal goat block (NGB, 10% Normal Goat Serum, Sigma, 1% Blocking powder, Perkin Elmer, PBS/T) for 30 minutes at room temperature. Primary antibodies (Rabbit anti-laminin, 1:5000, Abcam; GDF8_086, 50 μg/mL) were diluted in NGB and applied to sections overnight at 4°C. Sections were washed 3 times with PBS/T, and then incubated in secondary antibodies (Alexa Fluor 488 conjugated Goat anti-Rabbit, 1:1000, Invitrogen; Alexa Fluor 594 conjugated Goat anti-Human IgG FCϒ, 1:500, Jackson ImmunoResearch) diluted in NGB for 1 h. Sections were then incubated in 350 nM DAPI (Thermo), PBS/T for 5 minutes, washed twice with PBS/T, and then mounted with Vectashield (Vector Laboratories). For recombinant protein absorption experiments, 50 μg/mL GDF8_086 was incubated overnight alone or with 10x molar excess of either murine proGDF8 or proGDF11 in NGB, and then used as primary antibody. Fluorescent images were captured with a Leica DM4 B equipped with 40x/.80 Fluotar objective using Leica Application Suite X software. Images were then processed with Fiji^2^.

*Western blot from homogenized murine muscle and serum*

Frozen muscle tissue was pulverized in tissueTUBEs using a Covaris cryoPREP 02 impactor. Muscle lysates were prepared by taking approximately 50 µg pulverized muscle into a 1.5 ml microcentrifuge tube and immediately adding 400 µl T-PER lysis buffer (ThermoFisher) supplemented with 1X HALT protease/phosphatase inhibitor (ThermoFisher) and 1X Universal Nuclease (Pierce). Muscle was further disrupted using a disposable plastic pestle (Bio-Plas) and then by pipetting up and down several times through a large bore pipet tip. After 1 hour incubation at 4⁰C with constant rotation to allow complete lysis, muscle lysates were spun at 10,000g for 10 minutes to pellet undigested tissue debris and the un-pelleted fraction was removed to a new microcentrifuge tube. Protein concentration of the muscle lysates was determined by BCA assay (Pierce) and samples were normalized to 0.5-3 mg/ml by adding additional T-PER (w/ protease/phosphatase inhibitor). Lysates were then aliquoted for storage at -80⁰C or analyzed immediately by gel electrophoresis. Lysates were frozen/thawed no more than twice.

For western blot analysis, muscle lysates (5-30 µg) or 1:10 dilutions of serum or plasma (in PBS) were diluted in Laemlli Sample Buffer (Bio-Rad). Unless specifically indicated, all westerns were performed under reducing conditions by adding β-mercaptoethanol. Samples were denatured at 95⁰C for 5 minutes immediately prior to loading onto Any kD Mini-PROTEAN TGX Stain-Free Gels (Bio-Rad). After electrophoresis, the Stain-Free dye was activated by exposing the gel to UV light (302nm) for 5 minutes followed by blotting onto Immun-Blot Low Fluorescence PVDF membrane (Bio-Rad) using a TransBlot Turbo (Bio-Rad) gel transfer system.

Blotted membranes were blocked for 1 hour at room temperature in 1% polyvinylpyrrolidone in TBS + 0.1% Tween 20 (TBST). Antibody incubation was carried out overnight at 4⁰C in blocking buffer. Antibodies used: anti-myostatin prodomain – AF1539 (R&D Systems) used at 1:2000 dilution; anti-mature Myostatin – ab124621 (Abcam) used at 1:1000 dilution. After washing in TBST, membranes were incubated with HRP conjugated secondary antibody (1:5,000 anti-sheep or anti-goat, R&D Systems) for 1 hour at room temperature, washed in TBST. HRP activity was detected by chemiluminescence (Bio-Rad Clarity Western ECL Substrate) and imaged on an Azure c600 Gel imaging system (Azure Biosystems). After detection, equal loading between lanes was confirmed by Ponceau S staining. For quantitative fluorescent western blots, HRP conjugated secondary antibody was replaced with AlexaFluor680-conjugated anti-sheep or anti-goat (1:20,000, Jackson Immunoresearch) and quantified by near-IR imaging (Azure c600, Azure Biosystems).

*ELISA binding assay*

Biotinylated recombinant myostatin or GDF11 proteins (2 μg/mL) were added to BSA-blocked 96 well assay plates coated with 1 mg/mL NeutrAvidin Biotin-Binding Protein (Pierce). Following incubation, plates were washed three times with TBS (Tris-Buffered Saline; 50 mM Tris-Cl, 150 mM NaCl, pH 7.6) with 0.05% Tween-20. Test antibodies were then incubated at 1 μg/mL at room temperature for 1 hour, and the plates were again washed three times in wash buffer. Jackson ImmunoResearch Laboratories Peroxidase Affinipure Goat α-human FCγ Fragment Specific (1:25,000 dilution) was then added to the assay wells, and following a 1 hour incubation plates were washed three times in wash buffer. Luminescence signal was determined with the SuperSignal ELISA Femto Substrate (Pierce) according to manufacturer’s instructions. All experimental samples were assayed in triplicate. All averaged sample values above the calculated 3x standard deviation threshold (per antigen) were designated as hits.

*Epitope Binning*

Epitope binning data was generated using the Octet Qke biolayer interferometry system. For epitope binning against latent myostatin, 25 nM of biotinylated latent myostatin was used to coat a streptavidin sensor in 1x kinetics buffer (FortéBio) for three minutes. The immobilized latent myostatin was then presented with a set of up to eight different antibodies at 100 nM in 1x kinetics buffer and incubated for five minutes. After the five minute incubation/saturation with the first antibody, the sensors were re-presented with 100 nM of the first antibody and additionally presented with a second antibody at 100 nM. In this manner, all antibodies that bound to latent myostatin were evaluated for cross blocking/epitope binning.

For epitope binning against the mature GDF8 growth factor, the growth factor was immobilized to amine reactive tips. Tips were equilibrated in water for approximately 10 minutes before beginning the experiment. The first step of the experiment was 1 additional minute of equilibration in water followed by 5 minutes of activation with EDC and sulfo-NHS according to the manufacturer’s specifications. Mature GDF8 growth factor was then loaded at 5 μg/mL in pH 5 acetate buffer for ten minutes. Incubation in quench buffer (FortéBio) was performed for 5 minutes before a two minute baseline in 1x kinetics buffer. After the surface was prepared in this manner, epitope binning of all mature GDF8 growth factor binding antibodies was performed as described above. However, ten minutes was allowed for saturation of antibody 1 followed by an additional ten minutes for antibody 1+2 binding. In this manner, all antibodies that bind to mature GDF8 growth factor were evaluated for cross blocking/epitope binning.

The responses (nanometer shift over the entire duration of the binding step) generated by the binding of a unique “second antibody” were compared after saturation with each of the other antibodies in the panel as the “first antibody.” Response values were normalized for each second antibody such that binding after “saturation” with a non-latent-myostatin-binding isotype control was defined as a response of 1 (unimpeded binding). The response generated by binding a “second antibody” after saturation with that same antibody was defined as zero (no binding/noise) because in this case the epitope of the antibody should already be occupied.

Normalized responses of antibody B = (Binding after antibody A – binding after antibody B)/(Binding after isotype control – binding after antibody B)

After a table of normalized responses was generated for each antibody binding after all of the other antibodies for both latent myostatin and the mature growth factor, the data was imported into R version 3.3.1 for generation of a dendogram using the pvclust library. Boundaries were drawn for clusters with approximately unbiased P > 0.95, corresponding with a 95% confidence level.

Supplementary Figures:

**
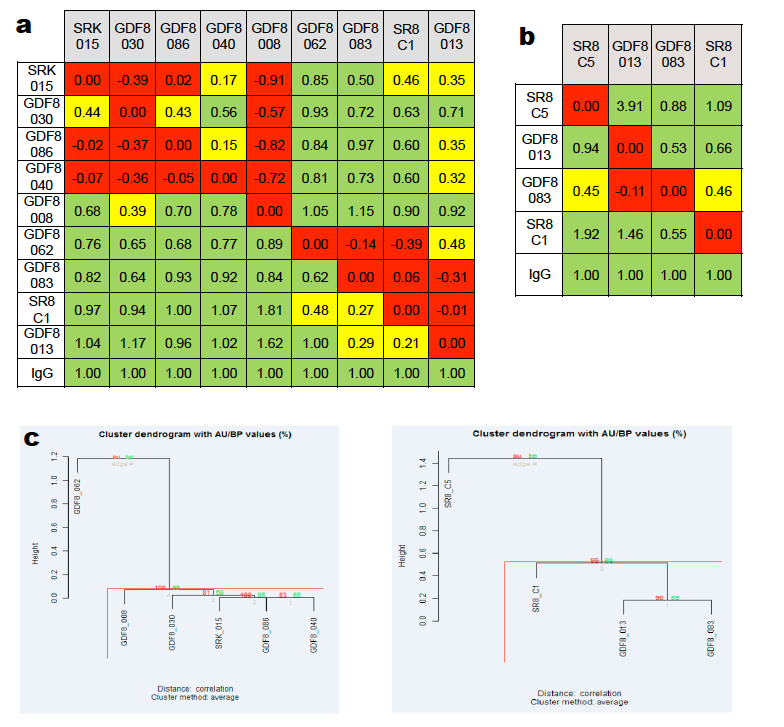
**

**Fig. S1. Cross-blocking experiments with a panel of Myostatin binding antibodies.** (**a**) Cross-blocking was carried out using a ForteBio BLI instrument, in which biotinylated latent murine myostatin was immobilized on a streptavidin-coated biosensor chip, and cross-blocking of antibodies was evaluated by sensor response. Normalized results are shown and colored as followed: red, binding of the first antibody blocked the binding of the second; yellow indicates a partial block, and green indicates no block. (**b**) Analogous experiments were performed for the mature growth factor. (**c**) Dendrogram analysis of epitope bins as evaluated by R. Assignment of epitope bins is presented in Table 1 of the main text.

**
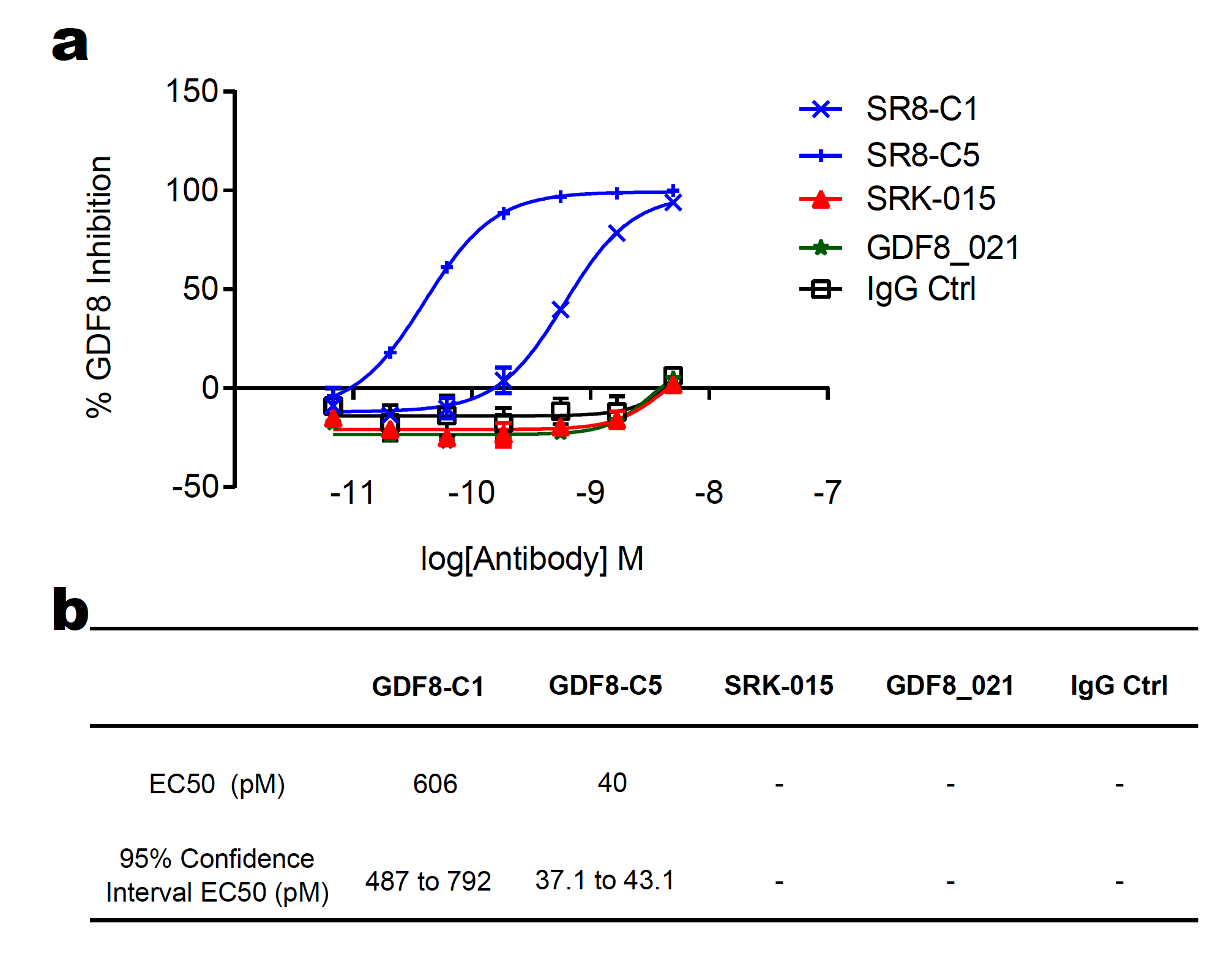
**

**Fig. S2.** **Control antibodies, but not Scholar Rock antibodies, block signaling of the mature GDF8 growth factor *in vitro*. (**a) SMAD-responsive reporter 293T cells were incubated with 2.5 ng/mL (0.1 nM mature dimer) of mature GDF8 growth factor preincubated with test antibodies (0.002 to 5 nM) in DMEM with 0.1% BSA for 6 hours. (b) EC50 values were calculated from a four parameter logistic curve. A – indicates the data were unable to be fit.

**
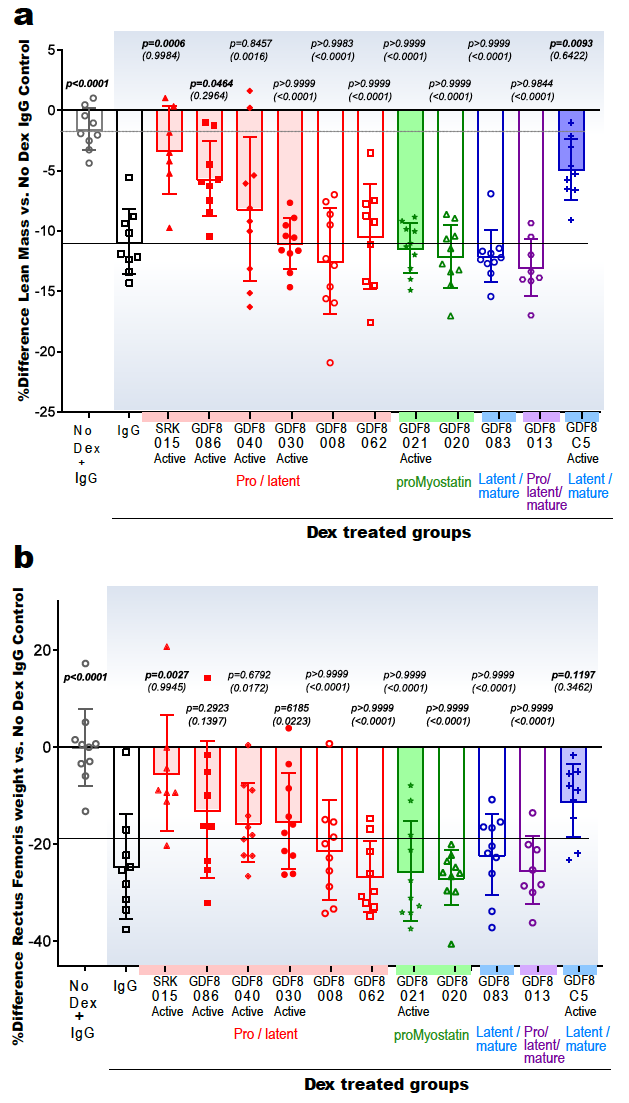
**

**Fig. S3. Evaluation of the efficacy of myostatin precursor-binding antibodies in a skeletal muscle atrophy model.** Animals were administered dexamethasone in their drinking water for two weeks, and dosed with 20 mg/kg of test antibodies once per week for two weeks (additional data in Fig. 2b). Plotted are the differences in lean mass by QNMR (a) and differences in rectus femoris weight (b) as compared to the mean of animals dosed with IgG control antibody (no dexamethasone administration, labeled as No Dex). Individual data points (n=8-10 animals, shown) along with means +/- standard deviations are shown. Group means were compared by one-way ANOVA followed by ANOVA followed by a Holm-Sidak test. Calculated P values for test article compared to the IgG control dosed in combination with dexamethasone are shown. Calculated P values compared to the group not administered dexamethasone (No Dex) are shown in parenthesis.


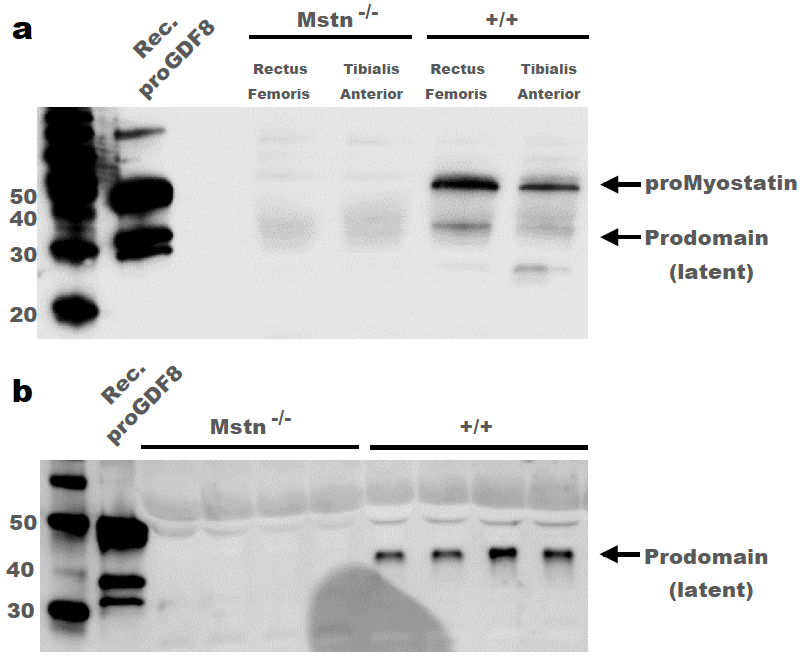


**Fig. S4. Predominance of proMyostatin in muscle and latent myostatin in serum. (a**) Western blot (primary antibody AF1539) analysis of myostatin levels in selected muscle homogenates from either wildtype (+/+) or MSTN-/- animals. Muscle and serum from MSTN-/- animals were utilized to verify specificity. (**b**) Analysis of myostatin precursor levels in serum. Recombinant proGDF8 is recombinant myostatin precursor material containing roughly equals amounts of proMyostatin and latent myostatin, thus serving as a marker for both species of the protein.

**
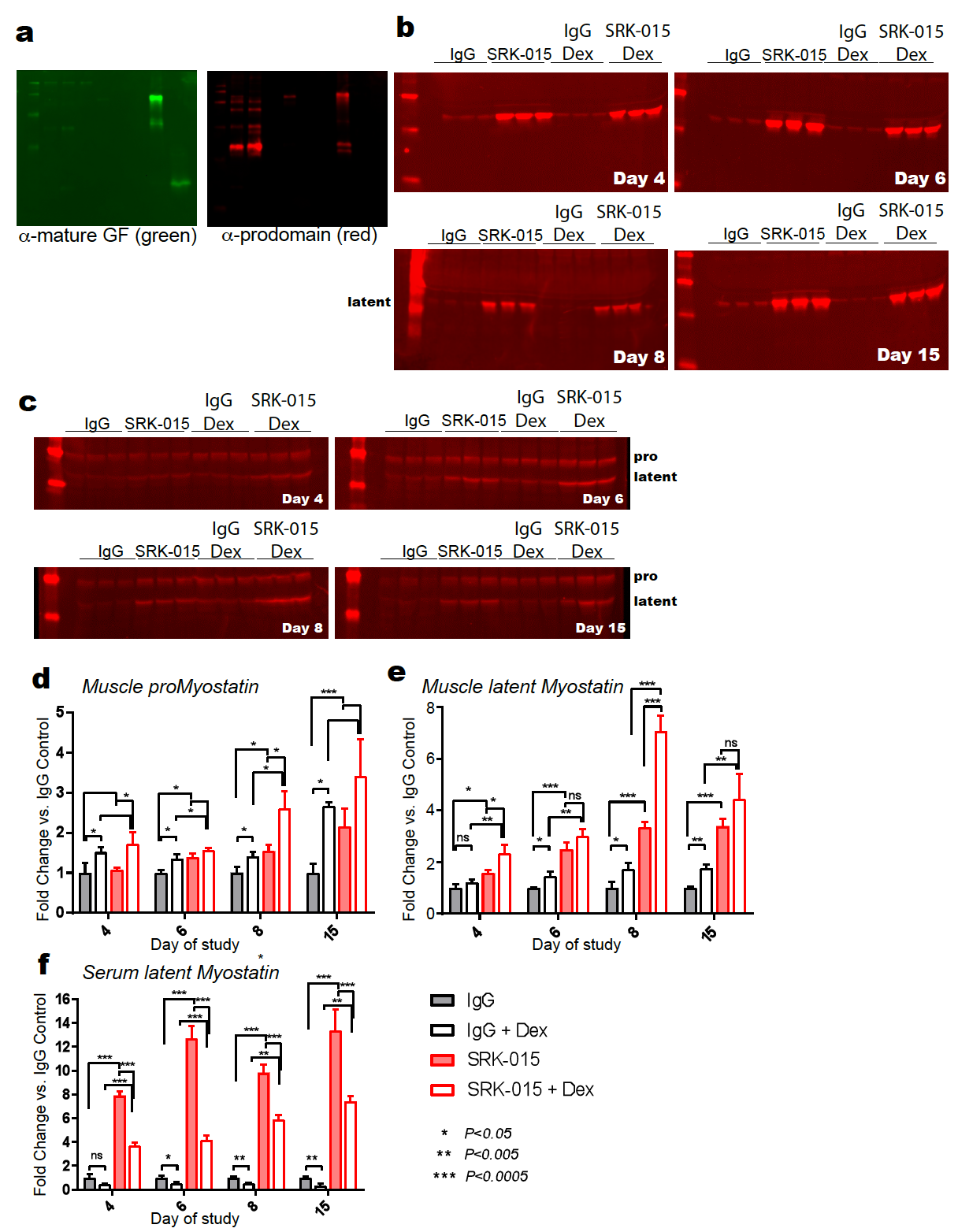
**

**Fig. S5. Representative images from fluorescent western blots. (**a) Red and green channels and raw data for fluorescent western blots overlaid in Fig. 3b. (b-**c)** Raw data quantitated in Fig. 4 (c-e). Images are from (**b**) serum and (**c**) Rectus femoris muscle lysates. (d-f) Quantitation of proMyostatin and latent myostatin levels in murine muscle and serum at 4, 6, 8, and 15 days following either dexamethasone or vehicle administration and concurrent treatment with either IgG control or SRK-015. The levels of proMyostatin and latent myostatin increased in rectus femoris muscle lysates taken from SRK-015 treated mice in both healthy and dexamethasone-treated animals. For all data presented (d-f), a minimum of three biological replicates were measured to generate the presented average values, and error bars on all graphs represent standard deviations. Statistical significance was determined by t test (two-tailed, homoscedastic).

**
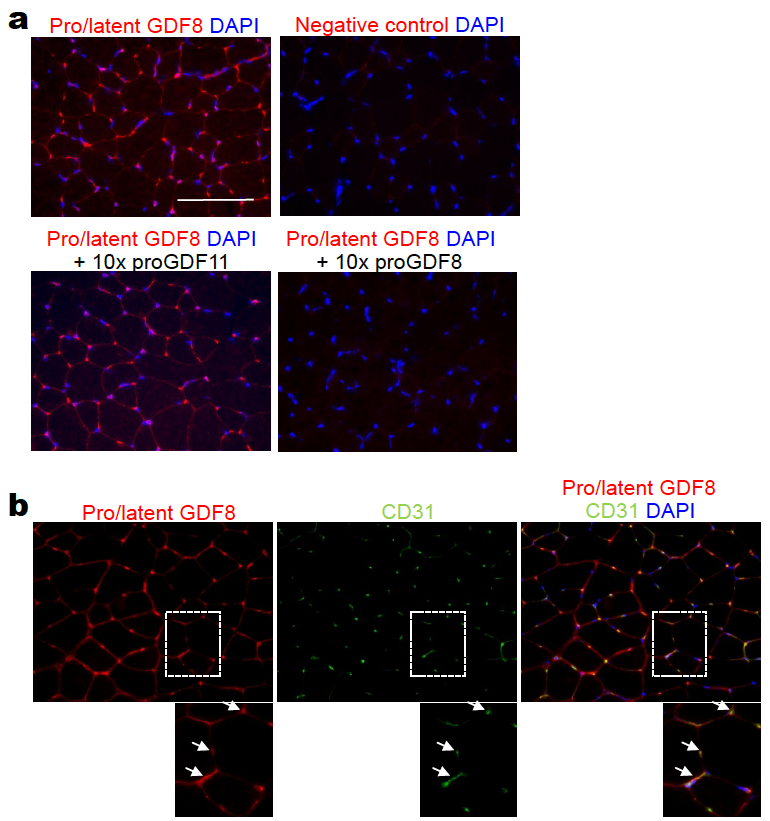
**

**Fig. S6. Specificity of immunofluorescence staining with the anti-pro/latent GDF8 antibody. (a)** Top: Cross sections of tibialis anterior muscle probed with anti-pro/latent GDF8 antibody (red; left, 100 μm scale bar) or non-specific targeting antibody, huNeg Negative Control (red; right), and counterstained with DAPI (blue). Bottom: Cross sections of tibialis anterior muscle probed with anti-pro/latent GDF8 antibody (red) that had been incubated in blocking buffer with 10-fold molar excess recombinant mouse proMyostatin (right), or incubated in blocking buffer with 10-fold molar excess recombinant mouse proGDF11 (left). Counterstained with DAPI (blue). (**b**) Co-staining of anti-pro/latent myostatin with the vascular marker CD31. All images are at the same magnification and scale.

**
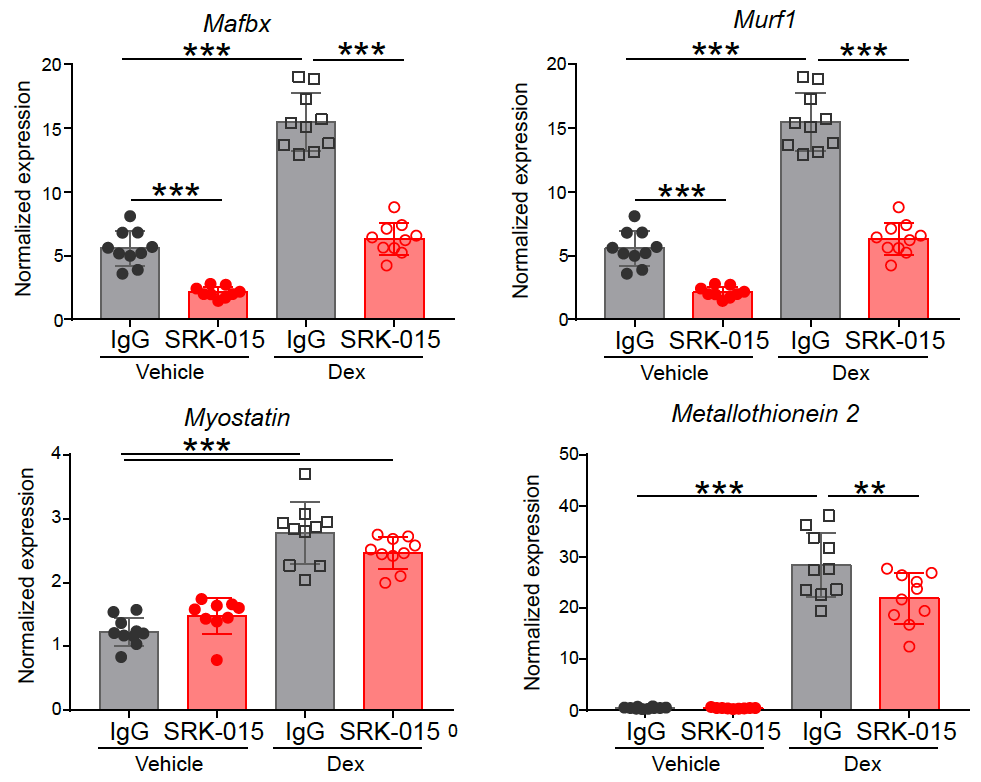
**

**Fig. S7. Gene expression was analyzed using the Quantigene Plex Assay.**  Animals were administered either vehicle (No Dex) or dexamethasone in their drinking water for two weeks, and given a single 20 mg/kg dose of test antibodies (SRK-015 or IgG control) at day 1 (Fig 4 b-e). On day 4 of the experiment gastrocnemius muscle was collected and gene expression (Mafbx, Murf1, Myostatin and Metallothionein 2) was analyzed by Quantigene Plex Assay. Expression of each gene was normalized to expression of two housekeepers, Rpl19 and Ppib. Data are mean ± SD. N=10. ***P<0.0001; **P=0.0056 by 2-way ANOVA with Tukey’s multiple comparison test.

**
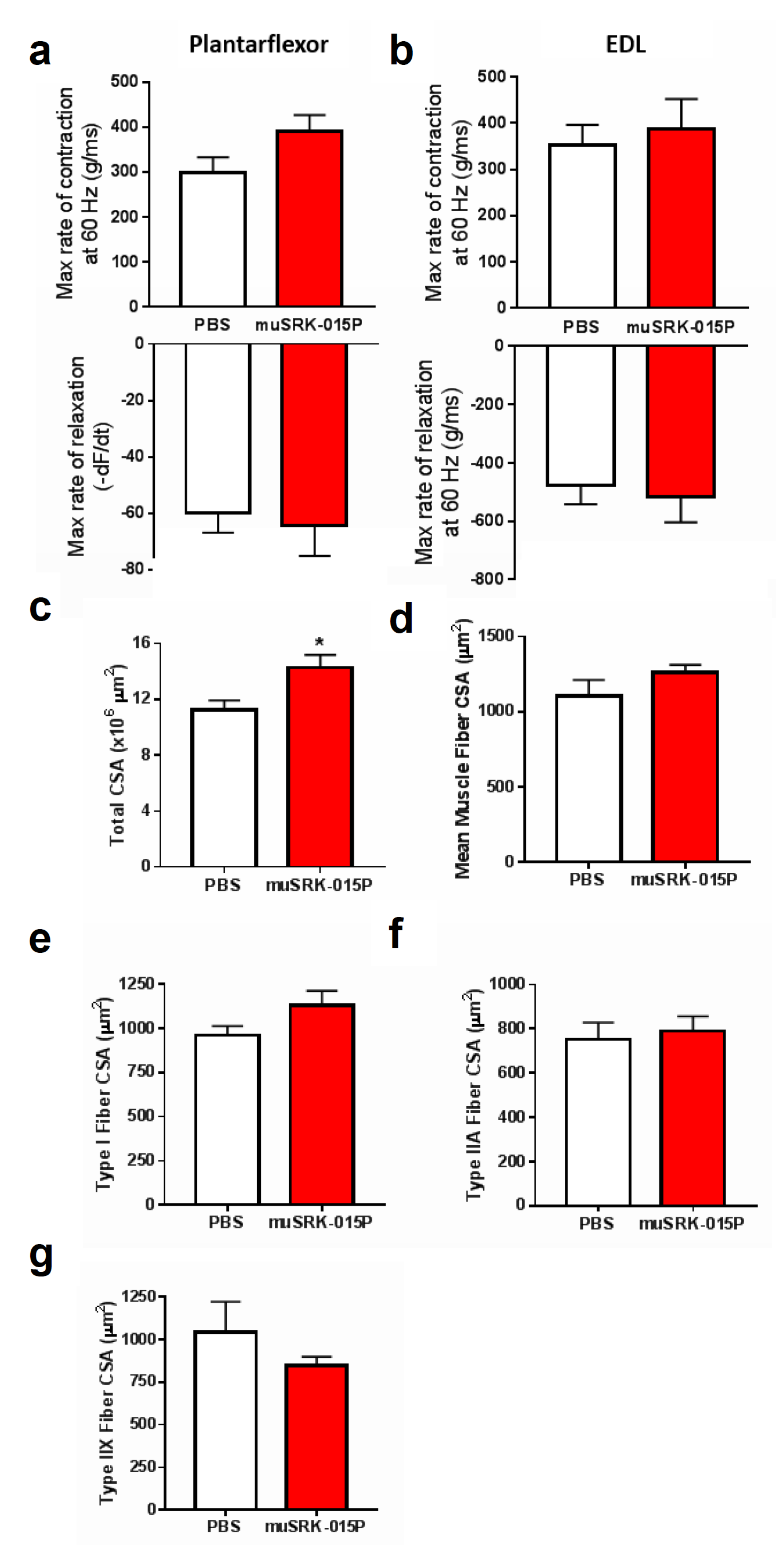

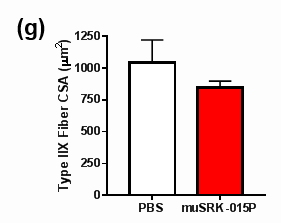
**

**Fig. S8. Impact of muSRK-015P treatment on muscle fiber hypertrophy and muscle performance.** Muscle function results in muSRK-015P treated mice after 4 weeks of treatment (additional analysis of experiment in Fig. 5). Maximum rates of contraction and relaxation of the plantarflexor group (**a**) and EDL (**b**). **(c-g)** Analysis of cross sectional area and fiber type from cryosectioned plantarflexor muscle group from animals treated with muSRK-015P for four weeks.

**Table S1. SRK-015P affinity and functional activity towards human/mouse proMyostatin. Affinity was measured with biolayer interferometry and functional activity was measured in human and mouse proMyostatin activation assays (200 nM human proMyostatin and 500 nM murine proMyostatin).**

|  | Functional Assay | Affinity |
| --- | --- | --- |
|  | EC50 (nM) | Kd (M) |
| Human | 274 | 4.76E-09 |
| Mouse | 839 | 1.10E-08 |

**Table S2. SRK-015 affinity and functional activity towards human/mouse proMyostatin.** SRK-015 affinity and functional activity towards human/mouse proMyostatin. Affinity was measured with biolayer interferometry and functional activity was measured in human and mouse proMyostatin activation assays (200 nM human proMyostatin and 500 nM murine proMyostatin).

|  | Functional Activity | Affinity |
| --- | --- | --- |
|  | EC50 (nM) | Kd (M) |
| Human | 248 | 3.42E-09 |
| Mouse | 714 | 8.39E-09 |

**Table S3. Affinities of SRK-015, muSRK-015 and muSRK-015P for murine proMyostatin**. Affinities were measured with biolayer interferometry.

|  | Kd (M) |
| --- | --- |
| muSRK-015 | 2.57E-09 |
| muSRK-015P | 2.88E-09 |
| SRK-015 | 8.35E-09 |

**Table S4. Calculated P values for Dexamethasone-induced atrophy study with once-weekly dosing for two weeks (data in Fig. 2b).**

|  | P *vs. IgG Control, No Dex* | P *vs. IgG Control, Dex* |
| --- | --- | --- |
| SRK-015 | >0.9999 | 0.0019 |
| GDF8_086 | 0.9856 | 0.0427 |
| GDF8_040 | 0.7896 | 0.1489 |
| GDF8_030 | 0.6351 | 0.2474 |
| GDF8_008 | 0.0092 | >0.9999 |
| GDF8_062 | <0.0001 | >0.9999 |
| GDF8_021 | 0.0017 | >0.9999 |
| GDF8_020 | 0.0026 | >0.9999 |
| GDF8_083 | 0.0061 | 0.9996 |
| GDF8_013 | 0.0450 | 0.9915 |
| GDF8-C5 | >0.9999 | 0.0012 |

1 Zhang, J. *et al.* Transient expression and purification of chimeric heavy chain antibodies. *Protein Expr Purif* **65**, 77-82, doi:10.1016/j.pep.2008.10.011 (2009).

2 Schindelin, J. *et al.* Fiji: an open-source platform for biological-image analysis. *Nat Methods* **9**, 676-682, doi:10.1038/nmeth.2019 (2012).
